# The decade of China’s football reform: Evolutionary characteristics, performance evaluation, and reflections and insights

**DOI:** 10.1371/journal.pone.0339264

**Published:** 2025-12-30

**Authors:** Zheng Li, Peng Du, Wanjin Deng, Dawei He, Xinyi Dong, Qifei Xia

**Affiliations:** 1 Faculty of Education, Shenzhen University, Shenzhen, China; 2 School of Physical Education, ShenZhen University, ShenZhen, China; 3 School of Physical Education, Ankang College, Ankang, China; Portugal Football School, Portuguese Football Federation, PORTUGAL

## Abstract

The year 2025 marks the tenth anniversary of the issuance of *the General Plan of Chinese Football Reform and Development*, and the deepening development of China’s football reform necessitates a robust policy framework for support. Quantitative research on policy texts enables an objective evaluation of the policy efficacy in China’s football reform. This study constructs a quantitative assessment and empirical analysis framework encompassing nine primary variables and 40 secondary variables by employing methods such as textual analysis, the Delphi method, content analysis, and the PMC index model. Combining the PMC index model with surface plots, it conducts a quantitative evaluation of ten representative national-level policy texts introduced by China. The results indicate that, the overall assessment of these ten policies is “good.” Among them, two policies (P1 and P3) are classified as exemplary policies, four (P4, P6, P7, and P8) are rated as good policies, and the remaining four are categorized as moderate policies. This suggests that while the policy design is generally scientifically sound and effective, there remains room for improvement in increasing the number of exemplary policies. The research conclusions suggest that exemplary policies exhibit characteristics of both localized top-level design and a leading-by-example effect; good policies encounter practical issues of coordinated policy design with localized deficiencies; and moderate policies still display governance phenomena of specialized institutional outputs coupled with mutual constraints. Therefore, the national strategy for China’s future football reform is characterized by longevity and gradualism, and there remains a lag in the implementation and actualization of policies. It is imperative to adopt a process-oriented mindset to achieve a substantive transformation from the advantages of policy texts to governance efficacy.

## 1 Introduction

As an iconic collective sport in the process of modern civilization, football, through its capacity for cultural identity construction and community mobilization, continues to fulfill multiple social functions such as social cohesion, economic development, and international exchanges [1]. Football is played in over 200 countries across the globe [2], and the development of football has proven to be of significant social importance, such as enhancing the national physical fitness, showcasing a country’s sports and cultural image, and promoting urban economic development [3]. In China, since the 18th National Congress of the Communist Party of China, General Secretary Xi Jinping has attached great importance to the development of football, regarding the revitalization of football as a crucial task in achieving the goal of building China into a leading sporting nation [4]. Ye [5] claims that developing and revitalizing China’s football cause is of great significance for improving the domestic football culture and advancing the reform of the “three major ball sports” (referring to football, basketball, and volleyball in Chinese sports context). Due to the repeated underperformance of China’s football team in international competitions and the declining commercial value of domestic football leagues, the Chinese government issued a landmark and programmatic football reform document, the “*General Plan of Chinese Football Reform and Development*” (hereinafter referred to as the “*Plan*”), in 2015, with the aim of revitalizing the football cause [6]. It is worth mentioning that 2025 marks the 10th anniversary of the release of the “*Plan*”. Conducting research on the policy text lineage derived from this plan is of significant importance in bridging the past and the future and it is beneficial for unveiling the institutional design logic of China’s football reform and facilitating the release of policy effectiveness in promoting football governance. However, looking back at over a decade of football reform in China, despite some progress made in the football sector due to this “*Plan*”, there are still discrepancies between policy and practice. Overall, the country is still facing issues such as a widening intergenerational gap in the competitive level of professional football [7], delays in advancing the goals of campus football [8], and a gradual decline in the development level of professional leagues [9].

Currently, the CPC Central Committee still insists on using football reform as a breakthrough point to coordinate and advance the reform of the “three major ball sports”, and has provided certain policy preferences to support the development of football [10]. Consequently, the academic community in China has placed increasing emphasis on research related to football reform. This is manifested in their exploration of sustainability issues in the football sector such as the recruitment of outstanding coaches and long-term reform of the youth training system [11], their analysis of the current development status and dilemmas of campus football in China [12], as well as their macro-level comparisons of the development of Chinese football from perspectives such as soft power and national identity [13]. Meanwhile, most studies on football policies also have predominantly focused on specific policy domains such as the development of campus football and the pilot zones for youth football reform [8,13]. However, Chinese scholars’ analyses of football reform mostly linger on the retrospective review of the reform process under the narrative of historical institutionalism and comparative studies of regional governance models in football [14,15], while the evaluation of reform effectiveness based on the paradigm of policymetrics remains relatively weak. Furthermore, the current international research trends in football reform, particularly within the realm of policy studies, have also directed attention to the performance of women’s football and the content of women’s football reform [16,17]. This has emerged as a crucial component of football reform initiatives in various countries. And recently, Chinese scholar Peng et al. (2025) [18] has begun to discuss the driving factors behind the phenomenon that Chinese Super League men’s clubs are required to incorporate women’s football teams in order to be eligible for league participation, the barriers to integration, and their implications for the future development of Chinese women’s football, but, on the whole, discussions among Chinese scholars regarding policies related to women’s football remain relatively limited. The objective assessment by Chen et al. [19], utilizing the Policy Modeling Consistency model(PMC model), of the applicability and effectiveness of China’s rural sports policies on rural sports development provides us with inspiration. It is equally valid to apply the PMC method for evaluating China’s football reform policies. The PMC is an analytical tool employed for multi-dimensional quantitative evaluation of policy texts, which can be used to “measure” the quality and internal consistency of policy documents. To this end, this study aims to conduct an empirical analysis of policies related to China’s football reform, especially encompassing content on women’s football reform and policies concerning youth campus football over the period from 2015 to 2025. By employing text analysis methods and constructing an evaluation system based on the PMC Index Model, this research will examine, from a quantitative research perspective, whether there exists an issue of institutional hollowness in the current football reform policies. Importantly, the purpose of our research is to effectively pinpoint selection biases in policy instruments and deficiencies in institutional design through quantitative evaluation and systematic analysis of policies. Specifically, this entails carrying forward and sustaining policies that have been appraised as “excellent” or “good”, refining and recalibrating those rated as “moderate”. By doing so, we aim to provide a solid decision-making basis for the policy orientation of China’s football reform in the next phase. Additionally, we strive to drive the transition of China’s football reform from strategic planning to concrete, measurable outcomes.

## 2 Literature review

### 2.1 Research on the PMC model in the field of sports policy

Frank et al. [20] argue that policy evaluation is a comprehensive, objective, and scientific process to assess a policy’s implementation effects and impacts, facilitating adjustments. Currently, scholars mainly use qualitative or quantitative methods for policy evaluation. Quantitative approaches, including the PMC Index Model, Difference-in-Differences (DID), and big data evaluation, are widely adopted across disciplines [21,22]. Policy modeling consistency (PMC) has become a mainstream method in policy evaluation due to its multi-level analysis, convenience, and objectivity. By utilizing policy text mining, PMC constructs a multi-dimensional evaluation indicator system. This approach overcomes the subjectivity of qualitative research and avoids the limitations of other mathematical and statistical methods, such as incomplete indicator systems. As a result, it has become a robust and prevalent methodology [23].

Sports policy research primarily focuses on sports policies introduced by the state (such as governments at the national level, voluntary or non-profit organizations at the national level, and national sports federations, etc.), and analyzes the impacts of these policies on certain areas of sports based on the political dimension of sports [24]. The term “sports system” frequently appears in the strategies and discourses of national governments aimed at promoting physical activities among the general population. However, “sports” is a practice that can, but does not always, yield positive health outcomes [25], and the same applies to sports policies. This necessitates the need for scholars from various countries to evaluate sports policies. Currently, policy research, including that in the sports field, has paid sufficient attention to the formulation and implementation aspects of policies, but relatively less attention to policy evaluation [26]. For instance, in the realm of school sports, Lindsey (2018) [27] utilized the punctuated equilibrium theory to analyze the patterns, implementation effectiveness, and changing trends in policy objectives of physical education and school sports policies in England after 2010. In the field of competitive sports, Funahashi et al. (2015) [28], considering the characteristic of “global competitive sports arms race” in the development of world sports, employed structural variance modeling to examine the socio-psychological mechanisms underlying public acceptance of elite sports policies. Joon-Ho Kang et al. (2025) [29] placed the study of sports policies within the framework of The Comprehensive Sport System (CSS), providing a comprehensive framework for integrated sports management and policy formulation that transcends traditional paradigms, and proposing normative development of sports policies from a systemic perspective. Nevertheless, there remains a lack of evaluation regarding the promulgation or implementation effects of sports policies in the aforementioned sports policy research.In recent years, the PMC Index Model has gradually gained attention as a quantitative evaluation tool in sports policy research, yet it is still at the exploratory stage. In terms of research scenarios, the PMC model in sports is mainly applied to evaluate school sports policies [30,31], analyze public service policies for nationwide fitness programs [19], and optimize sports consumption policies [32]. Regarding policy selection, domestic scholars such as Shi et al. (2023) [33] focus on national-level policy texts, whereas Lindsey (2020) [27] concentrates on elite sports policies, and Funahashi (2015) [28] examines specific sports policy characteristics during exceptional periods. In terms of evaluation methodologies, both domestic and international scholars predominantly use single-dimensional PMC model indices, with fewer adopting composite approaches. For example, Xing (2022) [30] used the PMC-AE composite method to assess the integration of sports and education policies in China, while Shi (2023) [33] employed the TM-PMC composite approach to analyze the evolutionary trends in nationwide fitness public service policies.Additionally, some scholars have applied the PMC Index Model to the quality assessment of emerging digital public sports service policies [34].

In summary, the PMC model provides a scientific tool and perspective for quantitative research in sports policy. However, its methodological application remains relatively confined to a single dimension, and research in this area has seldom delved into the realm of competitive sports. Consequently, this study adopts a composite evaluation method that combines text mining with the PMC Index, focusing its research on the practical domain of football reform. The aim is to facilitate the transition of sports policy evaluation from textual refinement to enhanced implementation effectiveness.

### 2.2 Relevant research in the field of football reform

In earlier research, Houlihan [35] argued that football reform would be a pivotal issue in global sports governance, given football’s developmental ties to a nation’s comprehensive strength, socio-cultural environment, and public physical literacy and athletic quality. Sullivan et al. [36] discovered that the development of Chinese football is intertwined with China’s national construction efforts, representing a sport driven by political objectives. Consequently, the policy design for this sport is more detailed and holds greater significance compared to that of other sports. In recent years, following the implementation of major football reform policies in China, there has been a surge in academic discourse on football reform within China’s scholarly community. Regarding football governance reform pathways, Chen et al. [19] examined regional football governance experiences and proposed a “well-ordered division of labor” strategy for China’s reform. In youth training and talent cultivation, both domestic and international scholars have emphasized the crucial role of school physical education (particularly campus football) in football governance reform [37,38]. And Lee [39] highlighted the revolutionary impact of school-based youth football management on national football reform and development. This provides theoretical support for our subsequent selection of policies related to youth football development. In policy formulation and top-level design, domestic scholars have focused on policies for school physical education reform in the new era, such as football-education integration strategies [40] and the construction of characteristic campus football schools [41]. Some scholars have also explored the reform pathways for China’s football management system, encompassing the promotion of institutionalization within the Chinese Football Association and strategies to better facilitate Chinese football clubs in attracting social investment [42]. A crucial aspect of China’s football management system reform lies in effectively managing the government-business relationship [43]. This, too, offers significant reference points for our subsequent selection of relevant policies. In contrast, foreign scholars have produced relatively few direct research findings on the formulation and top-level design of football reform policies but they have delved more extensively into social issues within the football realm, such as the impact of social media on the popularization of mass football and the development of professional leagues, the naturalization of foreign players in Chinese football, and the current development status of women’s football in society [17,44,45]. These studies offer fresh perspectives for policymakers to consider when focusing on the future direction of football reforms. Nevertheless, football reform, in general, constitutes a multifaceted and intricate issue that encompasses youth training, league operations, and other dimensions. Current research still lacks comprehensive studies that analyze the current state of football reforms from a macro policy evaluation perspective, particularly systematic assessments of the implementation effects of policy reforms in the areas of professional football, social football, and youth football. Thus, there is an urgent need for research on the effectiveness of China’s football reform to focus on the holistic, continuous, and systematic nature of policies.

To harness the macro-level control and regulatory functions of policy evaluation tools in football reform and advance theoretical research on policy evaluations in competitive sports, this study collects authoritative football reform policies issued by the state from 2015 to 2025. Utilizing methods such as textual analysis, the Delphi method, and the PMC index model, it conducts a macro-level, comprehensive evaluation of football development reforms over the past decade. The objective is to proactively detect and address institutional gaps during policy implementation through preemptive evaluations, thereby supporting the modernization of China’s football governance system and facilitating the translation of China’s football reform from a strategic plan into actionable outcomes.

## 3 Research design

### 3.1 Research framework

**Fig 1:**
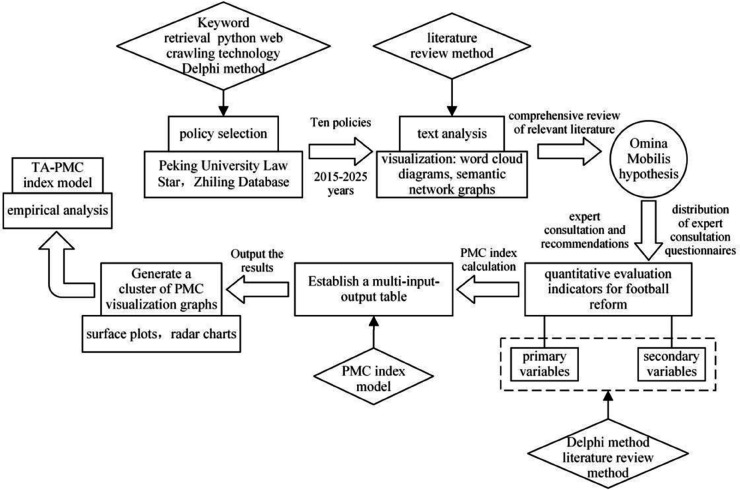
The evaluation process of constructing the TA-PMC index model.

Firstly, Peking University Legal Information Database (PKULAW), Zhiling Policy Database, and the official website of the General Administration of Sport of China were used as the database platforms for policy selection, and in conjunction with existing research findings [46] from previous studies, policy samples were collected in accordance with the principles of timeliness, relevance, and authority for sample screening. Secondly, policy text analysis was conducted using tools such as ROST CM6.0 and Nvivo12.0, encompassing word frequency statistics, word cloud diagrams, semantic network maps, and the annotation of fundamental content nodes within the policies. Subsequently, based on the results of the text analysis and combined with relevant literature on “football reform”, a quantitative evaluation index system for football reform policies was constructed, and expert questionnaires were developed encompassing both primary and secondary variable contents. Next, experts in the field of football reform were invited to score the questionnaires, and the content of the policy evaluation indicators was further refined based on the quantitative results from expert discussions and consultations. Finally, policy scoring grades were determined, and a multi-input-output analysis was conducted using the PMC index calculation formula, with visual graphic clusters presented based on these computational results. The design of this research plan essentially adheres to the following rationale for conducting an empirical analysis of the TA- PMC index model (Fig 1).

### 3.2 Policy selection and text source

We utilized the platforms mentioned above as the source repositories for policy samples. By employing keyword searches and Python web-crawling techniques with ’football reform’ and ’football work’ as keywords, we conducted searches on the selected databases or webpages over the time span from 2015 to 2025, adhering to the principles of timeliness and relevance. Secondly, during policy text cleaning and screening, the author exclusively included policy documents issued by national- level authoritative institutions such as the State Council, the General Administration of Sport of China, and the Ministry of Education, while excluding relevant local-level documents. Therefore, after determining the policy selection criteria, a preliminary screening yielded a total of 56 national-level policy texts on football reform that met the aforementioned standards. Given that the typical sample size range for policy research using the PMC index model is generally 8-12 items [33], the author, based on Ren et al.’s [14] conclusions on the periodization of football reform, the administrative hierarchy of the issuing institutions, and the quantitative consultation results from two rounds of expert interviews, ultimately selected 10 policies (Table 1) in accordance with the principles of authority and relevance.

**Table 1:** Representative Policies for Football Reform from 2015 to 2025.

Release Phase	Number	Document Title	Issuing Authority	Release Time
the period of reform with clearly defined objective (2015-2020)	P1	General Plan of Chinese Football Reform and Development	Chinese State Council	March, 2015
	P2	The Chinese Football Association’s Adjustment and Reform Plan	Chinese State Council	August, 2015
	P3	Medium and Long-term Development Plan for Chinese Football (2016-2050)	National Development and Reform Commission of China (NDRC)	April, 2016
	P4	2020 Action Plan of the Chinese Football Association	Chinese Football Association	January, 2017
	P5	Action Plan for the Construction of Eight Major Systems for National Youth Campus Football	Ministry of Education	September, 2020
	P6	Several Measures to Further Promote the Reform and Development of Football	Chinese Football Association	December, 2020
the period of deepening the content of reforms (2021-2025)	P7	Key Points of the National Youth Campus Football Work	Ministry of Education	April, 2022
	P8	Reform and Development Plan for Chinese Women’s Football (2022-2035)	General Administration of Sport of China	October, 2022
	P9	Implementation Opinions on the Reform and Development of Youth Football	General Administration of Sport of China	February, 2024
	P10	Administrative Measures for Social Football Events and Activities of the Chinese Football Association	Chinese Football Association	March, 2024

### 3.3 Research method

#### 3.3.1 Delphi method.

As a structured expert consultation methodology, the Delphi method holds significant value in prediction, decision-making, and problem-solving domains. In this study, the Delphi method plays a pivotal role in policy selection, deconstruction of evaluation dimensions, and weight assignment during PMC index calculation. Integrating expert consultation with the quantitative modeling process of the PMC index enhances the comprehensiveness and reliability of policy evaluation. The research engaged five experts in sports governance (four from academic institutions and one from an industry association) to provide recommendations on policy screening, variable selection, and the development of the evaluation index system. All the aforementioned experts were informed of the purpose of this study and signed informed consent forms. The experts’ information is presented in Table 2 (to protect the privacy of the experts, only their surnames are retained). These experts scored each indicator via paper-based questionnaires, and we determined the final evaluation criteria for the football reform policy texts based on their scoring results.

**Table 2:** Expert information form.

Expert Serial Number	Affiliation	Field of Expertise	Years of Experience
1	University	Football Reform	38
2	University	The Development of Competitive Sports in China	35
3	University	Campus Football in China	31
4	Association	Youth football competition	40
5	University	Sports Management	30

#### 3.3.2 Textual analysis method.

Textual analysis is a systematic approach to interpreting textual data, aiming to uncover the implicit meanings, structural characteristics, and sociocultural contexts embedded within the source material [47]. Prior to conducting textual analysis, researchers must first engage in text mining to collect the necessary raw data. Accordingly, this study employs ROST CM6.0 and Python’s Scrapy framework to design web crawlers for gathering relevant policy documents. Subsequently, tools such as Nvivo 12.0 and Excel are applied to perform word frequency analysis, generate word clouds, and construct semantic network graphs, thereby establishing a foundation for subsequent indicator system development and a comprehensive examination of policy texts.

#### 3.3.3 PMC index model.

PMC (Policy Modeling Consistency Index) index model is a policy quantitative assessment tool proposed by Estrada and other scholars based on the Omnia Mobilis hypothesis [48], which realizes the effectiveness measurement and optimization diagnosis of the policy text through the construction of a multi-dimensional policy evaluation system. The model construction and analysis process generally follows the steps below [49]: firstly, the deconstruction of evaluation dimensions, which includes the selection of primary and secondary variables, and the selection process can be based on the results of the text mining mentioned above; secondly, the construction of a multi-input-output table; and subsequently, the PMC indexes are calculated and PMC surface diagrams are drawn to show the policy evaluation effect. Given that the PMC index model can not only diagnose the systematic defects of existing policies, but also reveal the path of reform deepening through longitudinal comparison, it has applicability and theoretical significance in evaluating the implementation effect of policies in the field of football reform.

Before constructing a quantitative evaluation framework using the PMC index model for football reform, it is crucial to define primary (first-level) and secondary (second-level) variables. This process follows the principle of “data-driven analysis—textual guidance—expert validation”, ensuring alignment with policy text substance and scientific rigor in variable selection [48]. The data-driven phase employs text mining and analytical techniques, drawing on word frequency statistics and semantic network mapping to inform variable selection. The expert validation phase utilizes the Delphi method, involving three rounds of expert consultations to assess variable validity. Based on the hypothetical framework by Omnia Mobilis and three rounds of expert consultations, the Kendall’s coefficient of concordance (W) for each indicator was calculated at 0.673, indicating strong expert consensus [50]. This resulted in the establishment of nine primary and forty secondary variables (as detailed in Table 3). After constructing the policy evaluation indicator system, a multi-input-output table is generated to depict quantitative relationships among policy variables, using binary coding (1 for presence, 0 for absence) for sub- variables within each primary variable. It is noteworthy that the PMC index model generally employs the equal-weighting method for weight allocation (i.e., all first-level and second-level variables are assigned identical weights). This is because the core rationale of the PMC model is to objectively reflect the quality of policy texts based on their inherent content, with a particular emphasis on the balance and comprehensiveness of policy texts across various dimensions. However, experts are required to assign scores of 0 or 1 to the second - level indicators.

**Table 3:** Selection of policy evaluation indicator variables and their justification.

Primary variables	Serial number	Secondary Variables	Evaluation criteria for secondary variables	Reference point
*X*_1_ policy timeliness	*X*_1:1_	long-term	Whether the policy is planned for the long term (≥10 years), 1 if yes, 0 otherwise	Ruiz Estrada, 2022 [50]
	*X*_1:2_	mid-term	Whether the policy is a medium-term plan (5 to 10 years), 1 if yes, 0 otherwise	
	*X*_1:3_	short-term	Whether the policy is a short-term (<5 years) plan, 1 if yes, 0 otherwise	
*X*_2_ policy evaluation	*X*_2:1_	clarity	Whether the policy sets out clear objectives for reform, 1 if yes, 0 otherwise	Nkoua Nkuika et al., 2022 [51]
	*X*_2:2_	compatibility	Whether the policy fits in with the national sports development strategy, 1 if yes, 0 otherwise	
	*X*_2:3_	phased implementation	Whether the future implementation of the policy will be completed in phases, 1 if yes, 0 otherwise	
	*X*_2:4_	quantifiable	Whether the policy sets quantifiable targets, 1 if yes, 0 otherwise	
*X*_3_ incentive- constraint framework	*X*_3:1_	financial guarantee	Whether the policy has a clear funding plan and source, 1 if yes, 0 otherwise	Shi et al., 2023 [33]
	*X*_3:2_	regulatory penalties	Whether the policy specifies penalty provisions for non-compliance, 1 if yes, 0 otherwise	
	*X*_3:3_	incentive system	Whether the policy establishes positive incentives, 1 if yes, 0 otherwise	
	*X*_3:4_	oversight accountability	Whether the policy has a supervisory oversight component, 1 if yes, 0 otherwise	
	*X*_3:5_	talent incentives	Whether the policy has a talent incentive component, 1 if yes, 0 otherwise	
*X*_4_ policy domain	*X*_4:1_	professional football	Whether the policy addresses the area of professional football reform, 1 if yes, 0 otherwise	Based on semantic web mapping and word cloud mapping
	*X*_4:2_	campus football	Whether the policy addresses the area of campus footabll reform, 1 if yes, 0 otherwise	
	*X*_4:3_	social football	Whether the policy addresses the area of social football reform, 1 if yes, 0 otherwise	
	*X*_4:4_	women’s football	Whether the policy addresses the area of women’s football reform, 1 if yes, 0 otherwise	
*X*_5_ policy target audience	*X*_5:1_	football association	Whether the policy target addresses the reform of the Football Association, 1 if yes, 0 otherwise	Shi et al., 2023 [33] Based on semantic web mapping
	*X*_5:2_	football clubs	Whether the policy target addresses football club reforms, 1 if yes, 0 otherwise	
	*X*_5:3_	youth	Whether the policy target address youth reforms, 1 if yes, 0 otherwise	
	*X*_5:4_	manager	Whether the policy addresses manager reform, 1 if yes, 0 otherwise	
	*X*_5:5_	training camp	Whether the policy addresses training camp reform, 1 if yes, 0 otherwise	
*X*_6_ Policy perspectives	*X*_6:1_	macro	Whether the policy has a macro (national, international) perspective, 1 if yes, 0 otherwise	Hu et al., 2023 [32]
	*X*_6:2_	meso-level	Whether the policy is from a meso-level (provinces, municipalities and regions) perspective, 1 if yes, 0 otherwise	
	*X*_6:3_	micro	Whether the policy is from a micro (business, social group) perspective, 1 if yes, 0 otherwise	
*X*_7_ policy priority	*X*_7:1_	competition score	Whether the policy emphasizes the content of competition results, 1 if yes, 0 otherwise	Gong et al., 2025 [52] Based on semantic web mapping
	*X*_7:2_	cultivation of talent	Whether the policy emphasizes talent development content, 1 if yes, 0 otherwise	
	*X*_7:3_	mass participation	Whether the policy emphasizes mass participation content, 1 if yes, 0 otherwise	
	*X*_7:4_	institutional reform	Whether the policy emphasizes institutional reform elements, 1 if yes, 0 otherwise	
	*X*_7:5_	league adjustment	Whether the policy emphasizes the league adjustment element, 1 if yes, 0 otherwise	
	*X*_7:6_	Construction of football fields	Whether the policy emphasizes the construction of football fields, 1 if yes, 0 otherwise	
*X*_8_ policy characteristics	*X*_8:1_	announcement-oriented	Whether the policy is announcement-oriented or not, 1 if yes, 0 otherwise	Shi et al., 2024 [53]
	*X*_8:2_	predictive modeling	Whether the policy is predictive or not, 1 if yes, 0 otherwise	
	*X*_8:3_	supportive	Whether the policy has a support function, 1 if yes, 0 otherwise	
	*X*_8:4_	planning-oriented	Whether the policy is planned or not, 1 if yes, 0 otherwise	
	*X*_8:5_	Regulatory	Whether the policy involves regulation, 1 if yes, 0 otherwise	
*X*_9_ policy objectives	*X*_9:1_	competitive performance	Whether the policy aims at achieving improvements in competitive performance, 1 if yes, 0 otherwise	Ruiz Estrada, 2022 [50]
	*X*_9:2_	cultural atmosphere	Whether the policy aims to improve the ambience of the football environment, 1 if yes, 0 otherwise	
	*X*_9:3_	Robust mechanisms	Whether the policy include a goal related to establishing sound mechanisms 1 if yes, 0 otherwise	
	*X*_9:4_	Upgrading the population	Whether the policy has the capacity to achieve the goal of increasing the number of football pitches, 1 if yes, 0 otherwise	
	*X*_9:5_	Number of football Fields	Whether the policy includes an uplift in the number of football fields, 1 if yes, 0 otherwise	

At last, the study employs the measurement methodology proposed by Ruiz Estrada [50] to calculate the constructed evaluation framework and the aggregated policy outcomes, as demonstrated in Eq (1). Subsequently,the score of each first - level variable is that the arithmetic mean of the scores of all its subordinate second-level variables. Moreover, the quantitative assignment of second-level variables adopts a [0, 1] scoring method based on binary rules, as shown in Eqs (2) and (3). Finally,the PMC Index is calculated by summing up the scores of all nine first-level variables, thereby obtaining the PMC value for each Chinese football policy. These policies are then systematically categorized and evaluated according to the PMC Index grading criteria (see Table 4).

X∼N[0,1] (1)

X={XR:[0,1]} (2)

$$X_{i}\left(\sum_{j=0}^{n}\frac{X_{ij}}{T\left(X_{ij}\right)}\right)t=1,2,3,4,\cdots ,\infty$$ (3)

**Table 4:** Policy scoring grade criteria.

PMC Index Score	[0, 2.99]	(3, 4.99]	(5, 6.99]	(7, 10]
estimation	Poor	Moderate	Good	Excellent

Where, in Eq (3), “*t*” is a primary variable and “*j*” is a secondary variable.

$$PMC\;index\;=\;X_{1}\left(\sum_{i=1}^{3}\frac{X_{1i}}{3}\right)+X_{2}\left(\sum_{k=1}^{4}\frac{X_{2k}}{4}\right)+X_{3}\left(\sum_{m=1}^{5}\frac{X_{3m}}{5}\right)\;+X_{4}\left(\sum_{n=1}^{4}\frac{X_{4n}}{4}\right)+X_{5}\left(\sum_{o=1}^{5}\frac{X_{5o}}{5}\right)+X_{6}\left(\sum_{p=1}^{3}\frac{X_{6p}}{3}\right)\;+X_{7}\left(\sum_{q=1}^{6}\frac{X_{7q}}{6}\right)+X_{8}\left(\sum_{r=1}^{5}\frac{X_{8r}}{5}\right)+X_{9}\left(\sum_{v=1}^{5}\frac{X_{9v}}{5}\right)$$ (4)

Generally, the PMC index takes values in the range of [0, 9] under the binary assignment calculation method. Based on the values of *X*_1_ to *X*_9_ calculated by PMC index and construct a 3×3 PMC surface map for result presentation, as shown in Eq (5).

$$PMCsurface=[\begin{array}{ccc} X_{1} & X_{2} & X_{3}\\ X_{4} & X_{5} & X_{6}\\ X_{7} & X_{8} & X_{9} \end{array}]$$ (5)

Finally, with reference to Ruiz Estrada [50] and Pan et al. [54], who proposed the policy score evaluation criteria, the PMC score ranges from [0, 2.99] for poor, (3, 4.99] for moderate, (5, 6.99] for good, and (7, 10] for excellent in four levels of criteria (Table 4).

## 4 Results

### 4.1 Results of textual analysis

We conducted high-frequency word statistics and semantic network diagram construction for the 10 selected football reform policy texts, and then categorized them into periods based on “defining reform objectives” and “deepening reform content”. Data cleaning was performed by setting “stop words” to eliminate terms unrelated or irrelevant to the theme, following which the top 15 keywords related to the football reform theme were output in descending order of frequency (Table 5).

**Table 5:** Statistical results of word frequency for the top 15 most frequent words.

the period of reform with clearly defined objective 2015-2020			the period of deepening the content of reforms 2021-2025		
**Number**	**Keywords**	**Word frequency**	**Number**	**Keywords**	**Word frequency**
1	Football	951	1	Football	794
2	Reform	861	2	Reform	713
3	Development	460	3	Development	496
4	Football association	202	4	Adolescents	231
5	Objective	194	5	Campus	203
6	Management	138	6	Construction	168
7	Competition	143	7	Industry	121
8	System	118	8	Management	113
9	Organization	101	9	System	107
10	Cultivation	89	10	Talent	92
11	Talent	60	11	Market	63
12	National team	47	12	Football association	42
13	Industry	44	13	Powerful nation	39
14	Construction	43	14	Women’s football	36
15	Market	30	15	Youth training	33

**Fig 2:**
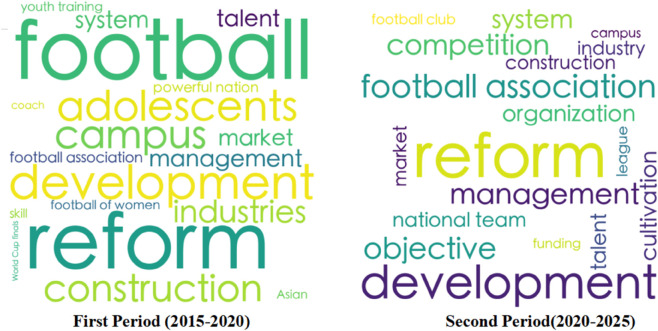
Word cloud of high-frequency terms.

Meanwhile, to more accurately depict the evolutionary trends during the first and second phases of football reform in China, the author conducted visual processing based on the word frequency statistics table, generating a high-frequency word cloud (Fig 2) that showcases the key characteristic changes in different periods over the more than a decade of reform.

**Fig 3:**
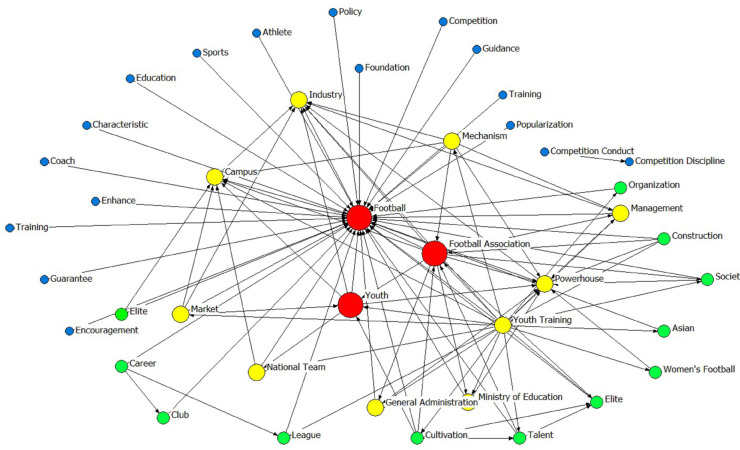
Semantic network map of 10 key words of football reform policies.

Furthermore, this study employed Nvivo 12.0 software to design nodes for each policy text and in conjunction with the high-frequency word statistics derived from ROST CM 6.0 constructed a semantic network map (Fig 3). In the semantic network model of this study, the associations between nodes and the structural characteristics of the entire network essentially depict the logical relationships and degrees of importance among high-frequency or central terms involved in various football reform policies. And the author linked various high-frequency thematic words from the football reform policies spanning 2015-2025 in the form of nodes and edges (network), with the size of the nodes indicating the strength of degree centrality. A stronger degree centrality implies greater importance of the node within the network and a higher number of connecting edges. Consequently, the structural relationships among high - frequency thematic words depicted in this semantic network diagram are based on the collinearity characteristics, semantic relations, and other features reflected in the policy text content that has been crawled. For instance, the term “football” appears with the highest frequency, and the first-tier high-frequency words centered around “football” are the Football Association and youth, which reflects that the content of football reform policies primarily revolves around the management of the Football Association and the development of youth football. Additionally, the semantic network map and the collinearity results of word frequencies can serve as a crucial reference for constructing evaluation indicators in the PMC index model.

The “*Plan*” issued in 2015 served as a programmatic document that guided China’s football reform from the first phase to the second phase, and established a macro-level institutional framework. Based on word frequency statistics and policy texts, the key themes and content evolution characteristics of China’s football reform over the past decade can be summarized as follows:

Firstly, China’s football reform has consistently centered on development as its core and sustainability as its goal. The reform is advanced around the notion of “development”, with policy design and practice exhibiting systematic, strategic, and sustainable qualities. During these two periods, the terms “reform” and “development” were mentioned most frequently, indicating that both programmatic and specialized reform plans are anchored in the concept of sustainable development. For instance, the *Medium and Long-term Development Plan for Chinese Football (2016-2050)* outlines a “three-step” strategic objective, encompassing multi-dimensional designs such as optimizing the management system, popularizing campus football, and enhancing professional leagues, with the aim of establishing a long-term mechanism for the sustainable development of the football cause.

Secondly, the construction of China’s football reform policy framework has evolved from laying a solid foundation to advancing in a coordinated manner. During the period of reform with clearly defined objectives (2015-2020), the reform focused on the fundamental restructuring of the management system, primarily taking “the de-administration of the Chinese Football Association (CFA)” and “the separation of regulatory and operational functions” as the core approaches. Subsequently, in the period of deepening the content of reforms, the frequency of words such as “youth”, “campus”, and “industry” has increased, signaling an in-depth development of the reform towards the integration of sports and education as well as industrial synergy. This aligns with the goal of coordinated development across various domains of China’s football reform, indicating that football reform is progressing continuously along the three-dimensional path of “government guidance-market-driven-social participation”.

Thirdly, the policy focus of China’s football reform centers on the innovation of the governance system and the restructuring of the youth training ecosystem. As revealed by the semantic network diagram, “Chinese Football Association” and “youth” serve as key thematic nodes, highlighting the strategic significance of institutional transformation and talent cultivation. In terms of the governance system, policies are dedicated to breaking through institutional barriers, primarily through measures such as the substantive reform of the Chinese Football Association (CFA) and to establish a mechanism for the separation of regulatory and operational functions [5]. Regarding talent strategy development, during the period of deepening reform, the national strategic emphasis has shifted towards the modernization of the youth training system. There has been an increased frequency of keywords like “youth”, “campus”, and “youth training”. Meanwhile, the thematic term “youth” forms close semantic associations with “campus” and “cultivation”, indicating that the national paradigm for football talent cultivation is evolving from a short-term, competition-oriented approach to a long-term, talent-nurturing mechanism.

Fourthly, there has been a notable increase in policy attention at the national level towards women’s football. Word frequency statistics show that since the promulgation of the “*Reform and Development Plan for Chinese Women’s Football (2022-2035)*”, the frequency of the term “women” in policy texts has surged significantly. During this same period, the focus of China’s football development has shifted from a singular emphasis on constructing a competitive system for men’s football to a diversified development landscape, forming a policy framework that concurrently advances men’s professional football, youth football, and women’s football.

### 4.2 Results of the PMC index model

#### 4.2.1 Results of constructing the multi-input-output table.

A multi-input-output table can be derived from the PMC index calculation formula, and the PMC index model’s evaluation results can be output based on the policy scoring hierarchy. Given that a total of nine evaluation indicators are established in this paper, the range of the PMC index is set from 0 to 9 rather than 0 to 10. Therefore, each original PMC index value (ranging from 0 to 9) is multiplied by 10/9 to convert it into the standard range of 0 to 10, and the results are shown in Table 6. As shown in Table 6, China’s football sector reforms over the past decade have generally shown marginal differentiation. The average PMC index for football reforms during 2015-2025 was 5.75 (above the benchmark of 5.00), and this places the overall performance in the “good” category. In the horizontal policy comparison, the 2015 “*Plan*” received an “excellent” rating with a score of 7.80, significantly outperforming the scores of other policies. A closely comparable score was achieved by *the Medium and Long-term Development Plan for Chinese Football (2016-2050)*, which garnered a high score of 7.61 and was also rated as “excellent”. The remaining policies received four “good” ratings and four “moderate” ratings, with both the proportion of “good” ratings and the proportion of “moderate” evaluations reaching 40%. This indicates that while the overall implementation effects of most policies are generally acceptable, there is still significant room for improvement among the policies, particularly in terms of the potential for introducing outstanding policies that could achieve breakthroughs.

**Table 6:** Table of evaluation results of ten football reform policies using the PMC index model.

Variables	P1	P2	P3	P4	P5	P6	P7	P8	P9	P10	Average
*X*_1_	0.33	0.33	0.33	0.33	0.33	0.33	0.33	0.33	0.33	0.33	0.17
*X*_2_	1.00	0.75	1.00	0.75	0.75	0.75	0.75	1.00	0.75	0.75	0.41
*X*_3_	1.00	0.60	1.00	0.60	0.20	0.40	0.60	0.60	0.40	0.40	0.29
*X*_4_	0.75	0.50	0.75	0.75	0.25	0.75	0.50	0.75	0.75	0.50	0.31
*X*_5_	0.80	0.40	1.00	0.60	0.40	0.80	0.40	0.80	0.60	0.60	0.32
*X*_6_	0.33	0.33	0.33	0.33	0.33	0.33	0.33	0.33	0.33	0.33	0.17
*X*_7_	1.00	0.33	0.83	0.67	0.33	0.83	0.50	0.83	0.33	0.33	0.30
*X*_8_	0.80	0.80	0.80	0.60	0.60	0.20	0.60	0.60	0.80	0.60	0.32
*X*_9_	1.00	0.40	0.80	0.40	0.40	0.40	0.60	0.60	0.60	0.60	0.30
TM-PMC Index	7.80	4.96	7.61	5.81	4.00	5.33	5.13	6.50	5.44	4.94	5.75
Ranking	1	8	2	4	10	6	7	3	5	9	-
Grade	excellence	moderate	excellence	good	moderate	good	good	good	moderate	moderate	good

In conclusion, based on the results of the PMC index and policy evaluation grade indicators, the ten policies are ranked in descending order as follows: P1 > P3 > P8 > P4 > P9 > P6 > P7 > P2 > P10 > P5. Meanwhile, P1 and P3 are classified as exemplary policies, P4, P6, P7, and P8 are categorized as good policies, and P2, P5, P9, and P10 are classified as moderate policies.

**Fig 4:**
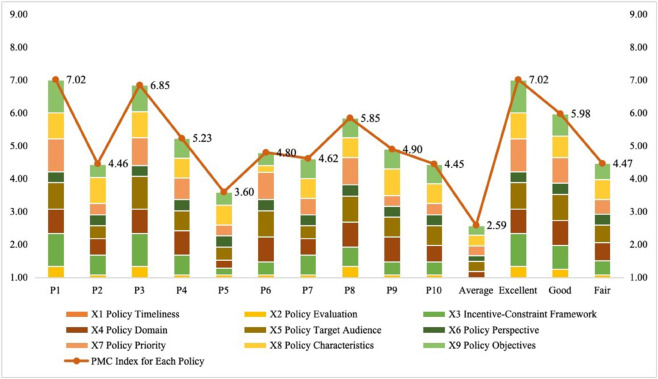
PMC index trend.

Additionally, by conducting a longitudinal comparison of policy implementation across different stages, it can be observed that during the period from 2015 to 2020, when reform objectives were clearly defined, the policy cluster (P1-P6) achieved a relatively high comprehensive score, with two of the policies receiving an excellent rating. During this phase, the “*Plan*” (2015) and the "*Medium- and Long-Term Development Plan for Chinese Football (2016-2050)*" ranked first and second, respectively, in terms of policy influence within the evaluation system. Among the policy clusters (P7-P10) during the period of deepening reform content (2020-2025), the “*Reform and Development Plan for Chinese Women’s Football (2022-2035)*” received the highest evaluation score of 6.50 points in this stage, ranking third in the overall ranking with a “good” rating. In contrast, the implementation effects of related policies during the same period appear relatively weak, with most of them receiving a “Moderate” rating. Furthermore, based on the trend chart of the PMC index (Fig 4), it is observed that the evaluation results of China’s football reform over the past decade have exhibited non-linear fluctuations and a downward trend, indicating a discrepancy between the implementation effects of the current policy system and the expectations of the general public.

#### 4.2.2 Results of constructing the PMC visualization graphics.

**Fig 5:**
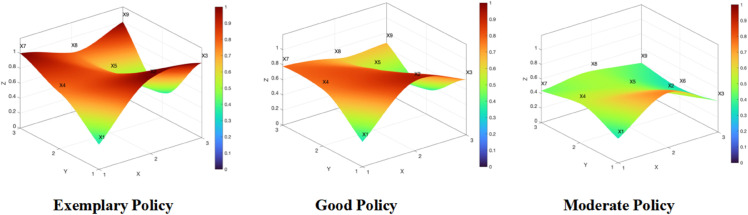
PMC surface chart of various types of policies.

The PMC surface diagram presented in this paper is constructed based on a multi - input - output table, incorporating axes for temporal evolution (X), policy attributes (Y), and performance evaluation (Z). Different score values for policy evaluations are distinguished through varying color blocks. In the diagram, higher scores are represented by convex peaks, while lower values are reflected as concave shapes. According to the PMC surface calculation formula, the scores ranging from *X*_1_ to *X*_9_ are input into the matrix, and this results in policies being categorized into exemplary (P1, P3), good (P4, P6, P7 and P8), and moderate (P2, P5, P9, and P10) types, with the PMC surface diagram shown in Fig 5.

**Fig 6:**
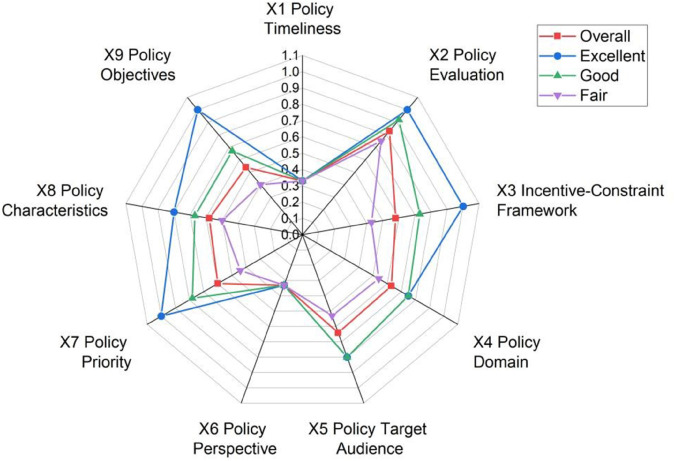
Mean-rank radar chart.

To assess horizontal disparities in the distribution of first-level variables across exemplary, good, and average policies, a “mean-rank” radar chart is plotted (Fig 6), which provides visual support for comparative analysis. It should be specifically noted that the statistical results of *X*_1_ (policy timeliness) and *X*_6_ (policy perspective) are unique. Regarding *X*_1_ policy timeliness, when analyzing a single policy text, it usually explicitly corresponds to only one specific time-effectiveness period. Experts will determine the time-effectiveness range that the policy explicitly corresponds to (for example, a “Five-Year Plan” corresponds to the medium-term). Therefore, only one secondary variable is assigned a value of 1, while the other two are assigned a value of 0. This results in all ten policies having a score of 0.33 on this dimension. However, this result is not meaningless; instead, it objectively reflects the singularity and focus of the policy in terms of time-effectiveness settings. If the time-effectiveness range cannot be determined, a value of 0 is assigned. Thus, it can be seen that all ten policies have a certain concept of planning duration. Similarly, *X*_6_ policy perspective examines the hierarchical range covered by the content of the policy text. If it is a macro-level policy, it cannot be a meso-or micro-level policy. Therefore, the scores of this variable are all 0.33. This scoring result is mainly due to the fact that the classic PMC index model uses the equal-weight method for weight allocation.

Given this, except for the two special indicators *X*_1_ (policy timeliness) and *X*_6_ (policy perspective), on the remaining first-level indicators, the scores of exemplary policies are higher than those of good policies, and the scores of good policies are higher than those of moderate policies, while the scores of moderate policies are below the average. Additionally, there is minimal variation in *X*_2_ (policy evaluation) scores across policy types, while significant differences are observed for *X*_9_ (policy objectives) and *X*_3_ (incentive-constraint framework). Overall, among the indicators excluding *X*_1_ and *X*_6_, *X*_2_ (policy evaluation) scores highest in the systematic evaluation (≈0.7 points), whereas *X*_9_ (policy objectives) scores lowest (≈0.5 points).

## 5 Discussion and conclusion

Through the building and computation of the PMC model for ten football reform policies, we can classify these ten policies into exemplary policies, good policies, and moderate policies. It has been found that the policies in the exemplary policy group are mainly the programmatic documents for football reform. These documents carry specific political tasks and objectives and simultaneously generate a “leading sheep effect”. Though it remains questionable whether this institutional effectiveness can be sustained, programmatic policies are the first to demonstrate institutional effectiveness during the reform process. Secondly, we have discovered that the good policy group mainly consists of specialized supporting policies. These policies play a crucial role in specific areas of football. In particular, the issuance of the *Reform and Development Plan for Chinese Women’s Football (2022-2035)* has resulted in the first dedicated reform document for Chinese women’s football with comprehensive policy content. Nevertheless, these policies perform poorly in terms of policy objective setting and the incentive-constraint framework, primarily because they are overly focused on a particular area, making it difficult to achieve comprehensive coverage of football reform. Additionally, the coordination effect among supporting policies is unsatisfactory. Finally, there is still significant room for improvement in the moderate policies. We have found that there are even some policy objective conflicts among the policy groups belonging to the moderate policies, which is mainly caused by the inconsistent policy objectives among different political entities. Moreover, policies belonging to this policy group also exhibit a certain degree of “tournament system inertia”, with some policy contents paying insufficient attention to the enhancement of the “soft power” of football reform. A detailed analysis of these types of policies will be discussed category by category, as follows:

### 5.1 Exemplary policy: Chinese-style top-level design and pioneering leader effect

As a crucial practice in the reform of the sports governance system with Chinese characteristics, the 2015 “*Plan*” and the *Medium and Long-term Development Plan for Chinese Football (2016-2050)* demonstrate the institutional effectiveness of top-level design and China-oriented reform design. For the first time in Chinese history, football development has been included as part of the national strategy [55]. This aligns with Liu’s [56] conclusion that “football reform must adhere to Chinese characteristics”. Moreover, these two policies not only reflect the strategic determination of the Central Committee of the Communist Party of China to “coordinate and harmonize nationwide actions” but also represent a top-down systematic design. Simultaneously, they embody the connection between Chinese football reform, national development, and political orientation as mentioned by Sullivan [36]. In other words, these two policies are consistent with both the long-term strategies of Chinese football reform and the political objectives of building a strong sports nation and revitalizing Chinese football [57]. From the perspective of outcomes, the “*Plan*” and *the Medium and Long-term Development Plan for Chinese Football (2016-2050)* stand out as the exemplary policy among football reform initiatives of the past decade, since their total scores of the PMC index rank first and second, respectively. As an exemplary policy, their content design comprehensively covers various evaluation dimensions, with each dimension scoring above the overall average. Notably, dimensions *X*_2_ (policy evaluation), *X*_3_ (incentive-constraint framework), *X*_7_ (policy priority), and *X*_9_ (policy objectives) all reached the theoretical maximum score of 1.00.

An in-depth analysis of the policy text reveals the following:

Firstly, in terms of the *X*_2_ dimension, this plan adopts a modern governance paradigm and sets specific, quantifiable objectives (such as establishing 20,000 football-featured schools by 2020, increasing the number to 50,000 by 2025, and constructing two national-level football training bases). It implements dynamic process control by dividing the reform into short-term, medium-term, and long-term goals, a feature that is more prominently demonstrated in Policy P3, namely *the Medium and Long-term Development Plan for Chinese Football (2016-2050)*. Policy P3 further elaborates on the three-step strategy for football reform by breaking it down into three more detailed phases and setting quantifiable targets for each phase. Notably, a common characteristic of these two policies is that they elevate policy content to the level of national political tasks, as evidenced by references to “fulfilling the football dreams of all Chinese people [36]”. Next, in the *X*_3_ dimension, the *Plan* combines incentive-based mechanisms with institutional constraints. It establishes a diversified financial guarantee system(e.g., a “special development fund for campus football”) and career planning/social security systems for athletes as incentives, while institutionalizing governance modernization (e.g., refining disciplinary frameworks and transparent legal oversight) to create a more comprehensive incentive-restraint framework than prior football reform policies.Furthermore, Policy P3 has introduced a series of detailed incentive policies, including supporting reward regulations, employment policies for football talents, and preferential tax and fee policies for football venues.

Moreover, in the *X*_7_ dimension, the “*Plan*” exhibits institutional innovations in football reform, primarily through pioneering management-level reforms, including the separation of government and social functions, as well as administrative and operational activities. These reforms transformed the Chinese Football Association from a public institution under the General Administration of Sport into a corporate entity, establishing a coordinated ecosystem with a three-tier league system and market entity development. Driven by these institutional changes, the “Chinese Football Administration Center” was abolished, facilitating the de-administratization and market-oriented evolution of football [43]. Additionally, the policy emphasizes youth talent development, systematically integrating youth training pathways with professional leagues to create a seamless channel from campus football to professional ranks, while promoting youth training camps and campus football popularization.

Lastly, in the *X*_9_ policy objectives dimension, the “*Plan*” and *the Medium and Long-term Development Plan for Chinese Football (2016-2050)* demonstrates scientific goal management. It divides football reform into short-term, medium-term, and long-term goals, avoiding short-term utilitarianism. This not only systematically maps out the strategic evolution of football reform but also converts strategic visions into an actionable evaluation system through the establishment of quantifiable indicators such as “a significant increase in the youth football population” and “the national men’s football team ranking among the top in Asia”. This approach clarifies the priorities and implementation pathways for each stage, thereby forming a bidirectional mapping mechanism between policy objectives and management practices.

**Fig 7:**
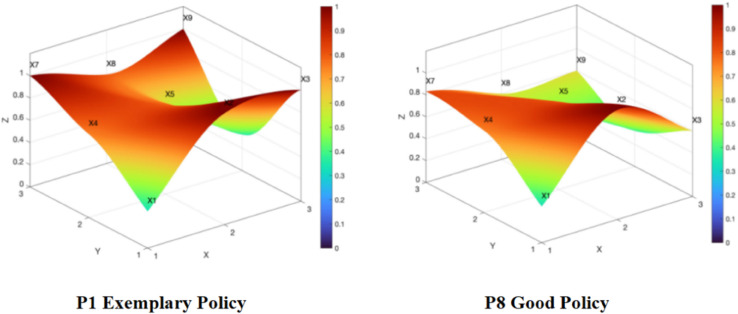
Comparison of PMC surface diagrams for exemplary policy and good policy.

An analysis of the aforementioned dimensions with outstanding performance can shed light on Wang’s [58] viewpoint regarding the initiatives for the development of China’s sports industry. Specifically, China’s sports development policies can be roughly divided into two categories: one emphasizes expanding the base, while the other focuses on enhancing the top-tier performance. In the context of football reform, the aforementioned two programmatic documents often serve as guiding policies that integrate and coordinate these two types of strategies. Consequently, their policy contents are more comprehensive, their objectives are more explicitly defined, and their political motivations are more pronounced, naturally embodying the hallmarks of exemplary policies. Meanwhile, based on the analysis of the results from the PMC index model, it can be observed that the top-level design of the 2015 football reform plan exhibits significant strategic value and institutional advantages. However, when comparing the exemplary policy (e.g., P1) with other good policies (e.g., P8) (as shown in Fig 7), it is evident that the “depression” degree of P8 is higher than that of P1. A greater degree of depression indicates a lower score. Moreover, the dark-colored segments in P1 are significantly more numerous than those in P8, reflecting that the average scores of various indicators in P1 are superior to those in P8. From this, we observe a pronounced “leading sheep effect” in the realm of football reform, highlighting disparities in content design and implementation effectiveness between systematic top-level design schemes and other related supporting policies. Furthermore, during the reform process, only the programmatic policies initially demonstrate institutional efficacy, but later on, these policies inevitably succumb to the law of diminishing policy effectiveness [59]. This may be attributed to the overly distant policy objectives set by these two types of policies (for instance, planning targets extending to 2050), which consequently constrain the overall implementation effectiveness of football reform in the future. Therefore, policymakers need to make dynamic adjustments, concurrently introducing corresponding short-term measures to complement long-term goals, and scientifically evaluating their performance throughout the entire policy lifecycle.

### 5.2 Good policy: Haphazardly coordinated policy design and partial deficiencies in content

A notable characteristic of good policies (such as P4, P6, P7 and P8) is their capacity to enhance policy effectiveness in specific domains or particular aspects [60]. Although these policies do not yet possess the comprehensive advantages of exemplary policies as a whole, they demonstrate strong innovation and practical value in certain key dimensions. Firstly, the score disparities among various dimensions of good policies are relatively small, and the range of the PMC curve’s depression remains within 0.4, indicating a relatively balanced scoring of policy elements and a certain degree of synergy among evaluation dimensions. Secondly, based on the evaluation results from the PMC index model (Table 6), while the scores of good policies in each evaluation dimension do not reach the theoretical maximum, breakthroughs have been achieved in the *X*_4_ policy domain and *X*_5_ policy target audience aspects, with these two indicators aligning with the scores of exemplary policies (approximately 0.8 points). In the *X*_4_ policy domain, policies P4, P6, and P8 all attain a score of 0.75, revealing strong inter-subsystem synergy within policies. A review of the policy texts reveals that this policy cluster exhibits strong coverage and integrity in key dimensions of football reform, primarily manifested in the following aspects:

First, there is continuity in professional football reform. Peng et al. [55] analyzed the two previous “decade-long” phases of China’s football development plans starting from 1993, whereas this paper examines the new decade beginning in 2015, thereby demonstrating the continuity of football reform policies. Particularly, it underscores that the reform of professional football in China cannot be achieved overnight and must focus on long-term structural reforms in areas such as management systems and professional leagues. Upon revisiting the policy texts, it can be observed that, for instance, the framework of “entity-based reform of the football association” established in P3’s *Medium and Long-term Development Plan for Chinese Football (2016-2050)* (Chapter IV, Section I) forms a governance iteration with the mechanism of separating administrative and operational functions in professional leagues outlined in P4’s *2020 Action Plan of the Chinese Football Association* (Chapter II). This reflects the mutual synergy and policy continuity among occupational football reform policies. Such characteristics are mirrored in Japan’s professional football reform system. And China’s professional football reform, particularly the development of its clubs, along with Japan’s professional football reform and club development, have provided certain referential insights for China [61].

Second, there is coherence in the content of campus football training. For instance, the “four-tier league system for campus football” proposed in P7 *Key Points of the National Youth Campus Football Work* (Chapter IV, Section II) achieves seamless integration across all age groups with the U-series youth training selection system constructed in P8’s *Reform and Development Plan for Chinese Women’s Football (2022-2035)* (Chapter II, Section II). This achievement is attributable to the implementation of “*Plan*” and the promulgation of various youth football initiatives. As a result, China’s campus football has gradually achieved an integrated and seamless connection between “learning, training, and competition” [62]. Meanwhile, a systematic pathway for the cultivation and development of football talents has been gradually established, as exemplified by the “one-stop” training system, guaranteed academic progression, and elite training programs (such as the establishment of the “Starry Sky” training camps). Similar to football reforms in other countries, the cultivation of youth football in China has emerged as a focal point, with a dedicated effort to refine the training of young football players. However, this endeavor is by no means straightforward. Some youth football promotion plans introduced by national sports administrations may conflict with the principles of holistic development and the integration of sports and education advocated by China’s Ministry of Education. Additionally, within society, families tend to be more inclined to support their children in focusing wholeheartedly on academic pursuits rather than fully engaging in football training [8,63]. Consequently, the varying social and cultural attitudes towards football in China, coupled with the divergent interest objectives of different policy stakeholders, have, to a certain extent, impeded the effective realization and impact of youth football policies.

Third, there are innovative pathways for specialized reform in women’s football. Prior to this, China had not implemented a systematic women’s football reform policy that had been elevated to the level of a national strategy, whereas scholars in other countries had already been engaged in discussions on how elite sports policies could enhance the international competitiveness and performance of women’s football [64]. In other words, countries with a more robust football development trajectory also place significant emphasis on the advancement of women’s football and the enactment of related policies [16]. Notably, China’s delayed introduction of a women’s football reform policy until 2022 may be intertwined with the underperformance and lackluster development of men’s football, which has led the nation to channel greater resources into the construction of men’s football. Nevertheless, this women’s football reform policy has drawn on the strengths of previously implemented policies, thereby earning high praise for its content. *Reform and Development Plan for Chinese Women’s Football (2022-2035)* formulates targeted deployments for the development and reform of women’s football, particularly by establishing institutional mechanisms for the coordinated development of regional women’s football and a U-series youth training pyramid system, thereby forging a novel pathway for the specialized development of women’s football. Consequently, this policy cluster achieves content complementarity and mutual synergy in the *X*_4_ policy domain. Despite variations in the scores of policy indicators in the *X*_5_ policy audience target aspect, an interdependent relationship among policies still forms, exhibiting characteristics of an “umbrella policy structure” [65].

Despite the fact that the PMC index scores of good policies across various dimensions surpass the overall average, there still exist “gaps” in the content design of this policy cluster. An analysis of the primary policy indicators reveals that, compared to exemplary policies, good policies demonstrate disparities in terms of the strategic orientation clarity of *X*_9_ policy objectives (around 0.7 points) and the systemic construction of *X*_3_ incentive-constraint framework (around 0.7 points). A deeper exploration into the policy texts indicates that, in evaluating the completeness and comprehensiveness of the *X*_9_ objective design dimension, only P6’s *Several Measures to Further Promote the Reform and Development of Football* has established a relatively systematic strategic objective framework. This document encompasses reform objectives covering competitive performance enhancement, football culture shaping, management mechanism adjustment, venue and facility construction, and football population popularization. In contrast, although P8’s *Reform and Development Plan for Chinese Women’s Football (2022-2035)* showcases breadth in objectives within areas such as competitive team building and youth training mechanism innovation, the absence of quantitative venue indicators in the infrastructure dimension restricts the multidimensional assessment of its policy objective design [16]. This design is intrinsically linked to its policy orientation focusing on gender-specific characteristics. As a specialized supporting policy, P4’s *2020 Action Plan of the Chinese Football Association* exhibits functional limitations in objective setting. Through qualitative analysis using Nvivo 12.0, it is evident that 48.7% of the coding nodes in this document are concentrated on talent gradient cultivation (28.1%) and the construction of the competition system (20.6%). There is a lack of indicators concerning the cultivation of a “football culture atmosphere” in terms of PMC quantitative indicators.

In sum, we found that the majority of policy clusters falling under the category of good policies are specialized supporting policies. Specifically, they are policies targeting specific areas of football reform, including women’s football, youth football, and policies related to the Chinese Football Association. In terms of policy objective descriptions, particularly those of secondary objectives, a flattened characteristic is evident, with an overall lack of specific hierarchical implementation path planning. This may be because setting overly specific strategic objectives could impose certain performance pressures on managers or athletes, as demonstrated in the conclusions of Zhang [66]. Additionally, this study has also observed that these policies neglect the cultivation of a football atmosphere. However, research indicates that countries with outstanding football performance often boast a very strong football atmosphere. Relevant studies [67,68] have confirmed that a favorable football atmosphere has a positive impact on the athletic performance of national teams, the growth in the number of fans, and talent cultivation in domestic leagues. Therefore, this study argues that in these supporting policies, there is a need to specifically introduce the strategic objective of “football culture cultivation” and implement corresponding incentive policies.

### 5.3 Moderate policy: Narrowly-focused institutional imposition and mutual restraints in content

Moderate policies often manifest as supplementary or specialized supporting policies for the early - stage institutional framework, which reveals systemic deficiencies in the design of policies related to football reform. Although this type of policy demonstrates refined institutional design within specific domains, it exhibits a preference for domain-specific policies [69]. Consequently, the design of each dimension tends to be specialized, resulting in scores for all dimensions being lower than the overall average. Based on the results, it can be observed that moderate policies only demonstrate a relative advantage in the *X*_2_ policy evaluation dimension (with a score of 0.75), which still falls below the overall mean for this dimension. Specifically, the four policies rated as “moderate” all achieved a score of 0.75 in the *X*_2_ policy evaluation, indicating a “clustering phenomenon” within this policy type in this particular dimension, where all these policies pay relatively good attention to the design of policy evaluation. This “clustering phenomenon” of policy indicators manifests itself in the following aspects:

Firstly, the policy texts exhibit homogenization characteristics in the design of policy evaluation. Whether it is policies targeting the reform of the football system or initiatives aimed at the popularization of campus football, there is a requirement to implement the contents of the “*Plan*”, demonstrating a strong alignment between policy texts and national strategies. Meanwhile, all moderate policies possess clear reform objectives. For instance, *the Chinese Football Association’s Adjustment and Reform Plan* focuses on streamlining the football management system and innovating the football management model, whereas *the Action Plan for the Construction of Eight Major Systems for National Youth Campus Football* emphasizes the consolidation and improvement of the institutional system for campus football work. Secondly, policymakers exhibit convergent yet differentiated attention in the evaluation design of this dimension. The fact that all policies score 0.75 in the *X*_2_ policy evaluation also suggests that there are deficiencies in certain aspects of their design, such as “clarity,” “alignment,” “phased implementation,” and “quantifiability.” For example, policies related to campus football, such as the Implementation Opinions on *the Reform and Development of Chinese Youth Football and the Action Plan* for the Construction of Eight Major Systems in National Youth Campus Football, both have clear objectives. However, the former, despite having a tiered implementation framework, lacks clear quantitative indicators, while the latter, although possessing quantitative goals, lacks a phased roadmap for decomposition. Therefore, although moderate policies exhibit relatively outstanding performance in the policy evaluation dimension, their failure to reach the theoretical maximum is attributed to their emphasis on refined evaluation of specific elements while neglecting systematic design. And the reason for this phenomenon lies in the fact that the content of these several types of specialized policies is typically oriented towards adjustments in specific areas. Nevertheless, relying solely on these policy contents makes it difficult to effectively translate the complex football reform into tangible outcomes. To achieve this goal, it is necessary to establish a policy evaluation system characterized by integrated design and integrated advancement.

Additionally, the strategic focus of China’s football governance system reform primarily centers on the structural adjustment of professional football and the construction of a youth talent development system within campus football. In this design process, moderate policies exhibit specialized supporting features concerning the aforementioned aspects, which, however, result in shortcomings in terms of the breadth and continuity of content design, failing to achieve comprehensive coverage in the evaluation of policy elements. Specifically, they score relatively low in dimensions such as *X*_3_ (Incentive-Constraint Framework) with 0.4 points, *X*_7_ (Policy Priority) with 0.4 points, and *X*_9_ (Policy Objectives) with 0.4 points. Indeed, the relevant documents of China’s football reform policies have still not been able to escape the influence of the inherent “tournament system inertia” in Chinese sports policies. This inertia has resulted in an excessive focus on quantifiable “hard indicators” in policy evaluation mechanisms and incentive orientations, such as “improving the world ranking of the Chinese men’s national football team” and “actively bidding to host the World Cup.” In contrast, the “soft indicators” within the football system, such as players’ technical and tactical proficiency and the social football atmosphere, have proven difficult to be effectively incorporated into the policy evaluation system. The concept of “ritual football” proposed by Li et al. [70] is reflected in these policy dimensions with low scores.

In terms of incentive-constraint framework, moderate policy clusters generally demonstrate an “asymmetric allocation of incentives and restraints” in institutional design. Only *the Action Plan for the Construction of Eight Major Systems for National Youth Campus Football* encompasses relatively specific measures such as “incentivizing innovation and establishing a campus football honor system” (Chapter II, Point 6) along with systematic safeguard measures (Chapter III). The remaining policy texts merely construct single-dimensional incentive mechanisms or solely include restraint and safeguard mechanisms, which can easily lead to a dilemma of “weak incentives and strong conflicts” during policy implementation. Furthermore, the *X*_7_ (Policy Priority) dimension reflects issues of “lack of coordination and mutual constraints”. An ideal policy design necessitates seamless collaboration between the sports system and the educational system. However, in reality, inherent disparities exist between the two systems in terms of objectives, resources, and evaluation frameworks. For instance, in the realm of campus football, *the Implementation Opinions on the Reform and Development of Youth Football*, spearheaded by the sports department, aims at the planning and design of competitive talent delivery, whereas *the Action Plan for the Construction of Eight Major Systems for National Youth Campus Football*, promoted by the education department, emphasizes curriculum design goals centered around fostering moral integrity, integrating sports and education. This leads to a dualistic conflict between a competitive focus and an educational orientation in policy priorities. This requires the sports authority and the educational department to jointly formulate a new policy framework for youth development, but the establishment of such a framework cannot be accomplished overnight [71]. Consequently, the contradiction between multidimensional objectives and limited resources in the *X*_9_ (Policy Objectives) dimension of moderate policies, specifically the conflicts between enhancing “competitive performance” and cultivating a “cultural atmosphere” in campus football, results in a convergence of contradictions between the *X*_9_ (Policy Objectives) and *X*_7_ (Policy Priority) dimensions. This, in turn, leads to similar phenomena in their PMC scores (0.4 points).

### 5.4 Conclusion

This study takes the football reform policies issued by national ministries and commissions from 2015 to 2025 as the research object, categorizing them into “the period of reform with clearly defined objective” and “the period of deepening the content of reforms” based on existing research findings. Through the application of the Delphi method, textual analysis, and the PMC index model, a quantitative performance evaluation and interpretation of China’s football development policies over the past decade have been conducted. The following key conclusions can be primarily drawn:

(1) China’s football reform embodies a policy-driven paradigm rooted in top-level design, with its evolutionary logic inherently stemming from the institutional endowments of the nationwide system. The *2015 General Plan of Chinese Football Reform and Development* elevated China’s football reform to a systematic project at the national strategic level, rather than a mere sports initiative. Consequently, China’s football reform represents a “battle to overcome formidable difficulties,” with its policy advancement process exhibiting characteristics of gradualism and longevity.

(2) China’s football reform primarily centers on the institutional reform of professional football (the separation of administrative and operational functions within the football association) and the development of campus football (the youth training system/popularization projects). However, there remains a lag in the implementation and actualization of football reform policies, particularly due to the conflicting objectives between the education sector’s advocacy for the integration of sports and education and the sports sector’s competitive orientation.

(3) The quantitative evaluation results of the policies indicate that, as a whole, the ten policies are rated as “Good.” Among them, the “*Plan*” and *Medium and Long-term Development Plan for Chinese Football (2016-2050)* are classified as exemplary policies, four others are rated as good, and four are considered moderate policies. This suggests that the overall policy design is scientifically sound and effective, yet there is still room for optimization. Meanwhile, based on the comprehensive results of the PMC index model, there is no risk of institutional hollowing out in the core of China’s football reform system. However, some supporting policies exhibit structural imbalances, which may lead to a lack of vigor during policy implementation. This could also be related to the “20-year talent cycle law” in competitive sports [72], which means that the effects of policies may not be immediately apparent. Furthermore, it reflects the complex challenge of balancing institutional design with realistic timelines in the reform process. Therefore, China’s football reform needs to proceed in a gradual and step-by-step manner, advancing the incremental institutional reconstruction of football reform. This approach should not only respect the particularities of China’s sports governance context but also adhere to the objective laws of football development, employing a process-oriented mindset to achieve a substantive transformation from the advantages of policy texts to governance efficacy.

Meanwhile, this study makes the following contributions: Firstly, during the calculation of the PMC index, we have moderately optimized the algorithm to enhance the scientificity and validity of the results. Specifically, we multiply the final result of the PMC index by 10/9 to ensure that the final range of the index can more scientifically align with the four categories of evaluation ranges. Secondly, we have systematically summarized the strengths and weaknesses of ten representative policies across various dimensions and uncovered the textual features of these representative policies, thereby providing theoretical guidance for future policy formulation and adjustment in China’s football reform. Finally, through this study, we can ascertain the overall evaluation of the policies issued during the new decade of China’s football reform (the overall evaluation is good). Additionally, it reaffirms that the new round of China’s football reform has indeed been elevated to a political task, carrying certain political connotations.

However, this study inevitably has certain limitations. These limitations primarily stem from the inherent constraints of the PMC index algorithm, which results in the binary assignment of some evaluation indicators, making it impossible to allocate weights reasonably. Future research can incorporate technologies such as Autoencoder (AE) to construct a PMC-AE model with data dimensionality reduction and nonlinear fusion capabilities, so as to better handle the complex relationships among variables. Moreover, the evaluation indicator system constructed in this study is difficult to achieve all-round coverage. After all, policy evaluation can be considered from multiple dimensions. Future research can further explore new evaluation dimensions based on this study, with the aim of conducting a more comprehensive and in-depth evaluation of football reform policies.

## Supporting information

S1 Appendix Expert survey questionnaire. (PDF)

S2 Source Data (Link) (10.6084/m9.figshare.30628091). (TAR)
