# Supplementary material for: The decade of China’s football reform: Evolutionary characteristics, performance evaluation, and reflections and insights
Source: PLoS One. 2025 Dec 30;20(12):e0339264. doi: 10.1371/journal.pone.0339264 (PMC12753081; doi:10.1371/journal.pone.0339264)
Supplement: S1 Appendix — (PDF) [file pone.0339264.s001.pdf]

Appendix 1

Research on the Evaluation of Chinese Football Reform Policies Based on the TA-PMC Model (First Round)

Dear Coaches/Experts:

Hello！

This study aims to screen and determine the core indicators for evaluating Chinese football reform policies through expert opinions, laying the foundation for subsequent analysis of policy evolution characteristics and efficacy. Your professional insights are crucial to this study, and we sincerely appreciate your support!

Now, please rate the importance of the indicators based on your actual situation and thoughts, and mark your scores in the corresponding options. If you have any new indicators or opinions, please also propose them; if there are no modifications, you may leave them blank.

Part I: Basic Information of Experts

1、Name：____

2、Gender：_____

3、Work Unit：_____

4、Years of Teaching or Training Experience：_____

5、Professional Title：_____

6、Research Direction：_____

Part II: Screening of Evaluation Indicators for Football Reform Policies

1、Instructions for Filling Out:

Please rate the importance of the evaluation indicators according to the following scale:

Very important	Important	Moderately important	Not important	Not at all important
5	4	3	2	1

2、Please rate the importance of the following indicators according to the above scale and fill in the "Importance Score" column. If you have any adjustment opinions on the following indicators, you may write them in the "Modification Suggestions" column. Thank you for your valuable opinions!

First-Level Indicators

Indicator Content	Very important	Important	Moderately important	Not important	Not at all important	Modification Suggestions
X1Policy Timeliness						
X2Policy Evaluation						
X3Incentives and Constraints						
X4Policy Domains						
X5 Policy Target Audiences						
X6 Policy Perspectives						
X7 Policy Priorities						
Additional First-Level Indicators						

Second-Level Indicators

Indicator Content	Very important	Important	Moderately important	Not important	Not at all important	Modification Suggestions
X1:1 long-term						
X1:2 medium-term						
X1:3 short-term						
X2:1 clarity						
X2:2compatibility						
X2:3phased implementation						
X2:4 quantifiable						
X3:1financial guarantee						
X3:2regulatory penalties						
X3:3incentive						

system						
X3:4oversight accountability						
X3:5talent incentives						
X4:1professional football						
X4:2campus football						
X4:3social football						
X5:1football association						
X5:2football club						
X5:3youth						
X5:4manager						
X5:5training camp						
X6:1macro						
X6:2meso-level						
X6:3micro						
X7:1competition Results						
X7:2cultivation of talent						
X7:3mass participation						
X7:4institutional reform						
X7:5league adjustment						
X7:6Construction of football fields						
Additional Second-Level Indicators						

*This questionnaire ends here. Please check if any items are missing.

*Thank you very much for your support and cooperation. Wish you a pleasant work and life!

Appendix 2

Research on the Evaluation of Chinese Football Reform Policies Based on the TA-PMC Model (First Round)

Dear Coaches/Experts:

Hello！

This study aims to screen and determine the core indicators for evaluating Chinese football reform policies through expert opinions, laying the foundation for subsequent analysis of policy evolution characteristics and efficacy. Your professional insights are crucial to this study, and we sincerely appreciate your support!

Now, please rate the importance of the indicators based on your actual situation and thoughts, and mark your scores in the corresponding options. If you have any new indicators or opinions, please also propose them; if there are no modifications, you may leave them blank.

Part I: Basic Information of Experts

1、Name：____

2、Gender：_____

3、Work Unit：_____

4、Years of Teaching or Training Experience：_____

5、Professional Title：_____

6、Research Direction：_____

Part II: Screening of Evaluation Indicators for Football Reform Policies

1、Instructions for Filling Out:

Please rate the importance of the evaluation indicators according to the following scale:

Very important	Important	Moderately important	Not important	Not at all important
5	4	3	2	1

2、Please rate the importance of the following indicators according to the above scale and fill in the "Importance Score" column. If you have any adjustment opinions on the following indicators, you may write them in the "Modification Suggestions" column. Thank you for your valuable opinions!

First-Level Indicators

Indicator Content	Very important	Important	Moderately important	Not important	Not at all important	Modification Suggestions
X1Policy Timeliness						
X2Policy Evaluation						
X3Incentives and Constraints						
X4Policy Domains						
X5 Policy Target Audiences						
X6 Policy Perspectives						
X7 Policy Priorities						
X8policy characteristics						
X9 policy objectives						
Additional First-Level Indicators						

Second-Level Indicators

Indicator Content	Very important	Important	Moderately important	Not important	Not at all important	Modification Suggestions
X1:1 long-term						
X1:2 medium-term						
X1:3 short-term						
X2:1 clarity						
X2:2compatibility						
X2:3phased implementation						

X2:4 quantifiable						
X3:1financial guarantee						
X3:2regulatory penalties						
X3:3incentive system						
X3:4oversight accountability						
X3:5talent incentives						
X4:1professional football						
X4:2campus football						
X4:3social football						
X5:1football association						
X5:2football club						
X5:3youth						
X5:4manager						
X5:5training camp						
X6:1macro						
X6:2meso-level						
X6:3micro						
X7:1competition Results						
X7:2cultivation of talent						
X7:3mass participation						
X7:4institutional reform						
X7:5league adjustment						
X7:6Construction of football fields						
X8:1announcement-oriented						
X8:2predictive modeling						
X8:3supportive						
X8:4planning-oriented						
X8:5regulatory						

X9:1competitive performance						
X9:2cultural atmosphere						
X9:3Number of football Fields						
Additional Second-Level Indicators						

*This questionnaire ends here. Please check if any items are missing.

*Thank you very much for your support and cooperation. Wish you a pleasant work and life!

Appendix 3

Research on the Evaluation of Chinese Football Reform Policies Based on the TA-PMC Model (Third Round)

Dear Coaches/Experts:

Hello！

This study aims to screen and determine the core indicators for evaluating Chinese football reform policies through expert opinions, laying the foundation for subsequent analysis of policy evolution characteristics and efficacy. Your professional insights are crucial to this study, and we sincerely appreciate your support!

Now, please rate the importance of the indicators based on your actual situation and thoughts, and mark your scores in the corresponding options. If you have any new indicators or opinions, please also propose them; if there are no modifications, you may leave them blank.

Part I: Basic Information of Experts

1、Name：____

2、Gender：_____

3、Work Unit：_____

4、Years of Teaching or Training Experience：_____

5、Professional Title：_____

6、Research Direction：_____

Part II: Screening of Evaluation Indicators for Football Reform Policies

1、Instructions for Filling Out:

Please rate the importance of the evaluation indicators according to the following scale:

Very important	Important	Moderately important	Not important	Not at all important
5	4	3	2	1

2、Please rate the importance of the following indicators according to the above scale and fill in the "Importance Score" column. If you have any adjustment opinions on the following indicators, you may write them in the "Modification Suggestions" column. Thank you for your valuable opinions!

First-Level Indicators

Indicator Content	Very important	Important	Moderately important	Not important	Not at all important	Modification Suggestions
X1Policy Timeliness						
X2Policy Evaluation						
X3Incentives and Constraints						
X4Policy Domains						
X5 Policy Target Audiences						
X6 Policy Perspectives						
X7 Policy Priorities						
X8policy characteristics						
X9 policy objectives						
Additional First-Level Indicators						

Second-Level Indicators

Indicator Content	Very important	Important	Moderately important	Not important	Not at all important	Modification Suggestions
X1:1 long-term						
X1:2 medium-term						
X1:3 short-term						
X2:1 clarity						
X2:2compatibility						
X2:3phased implementation						

X2:4 quantifiable						
X3:1financial guarantee						
X3:2regulatory penalties						
X3:3incentive system						
X3:4oversight accountability						
X3:5talent incentives						
X4:1professional football						
X4:2campus football						
X4:3social football						
X5:1football association						
X5:2football club						
X5:3youth						
X5:4manager						
X5:5training camp						
X6:1macro						
X6:2meso-level						
X6:3micro						
X7:1competition Results						
X7:2cultivation of talent						
X7:3mass participation						
X7:4institutional reform						
X7:5league adjustment						
X7:6Construction of football fields						
X8:1announcement-oriented						
X8:2predictive modeling						
X8:3supportive						
X8:4planning-oriented						
X8:5regulatory						

X9:1competitive performance						
X9:2cultural atmosphere						
X9:3Robust mechanisms						
X9:4Upgrading the population						
X9:5Number of football Fields						
Number of football Fields						
Additional Secondary Indicators						

*This questionnaire ends here. Please check if any items are missing.

*Thank you very much for your support and cooperation. Wish you a pleasant work and life!
