# Supplementary material for: The decade of China’s football reform: Evolutionary characteristics, performance evaluation, and reflections and insights
Source: PLoS One. 2025 Dec 30;20(12):e0339264. doi: 10.1371/journal.pone.0339264 (PMC12753081; doi:10.1371/journal.pone.0339264)
Supplement: S2 Source Data — (TAR) [file pone.0339264.s002.tar › Source Data/appendix.docx]

Appendix 1

Research on the Evaluation of Chinese Football Reform Policies Based on the TA-PMC Model (First Round)

Dear Coaches/Experts:

Hello！

This study aims to screen and determine the core indicators for evaluating Chinese football reform policies through expert opinions, laying the foundation for subsequent analysis of policy evolution characteristics and efficacy. Your professional insights are crucial to this study, and we sincerely appreciate your support!

Now, please rate the importance of the indicators based on your actual situation and thoughts, and mark your scores in the corresponding options. If you have any new indicators or opinions, please also propose them; if there are no modifications, you may leave them blank.

**Part I: Basic Information of Experts**

1、Name：____

2、Gender：_____

3、Work Unit：_____

4、Years of Teaching or Training Experience：_____

5、Professional Title：_____

6、Research Direction：_____

**Part II: Screening of Evaluation Indicators for Football Reform Policies**

1、Instructions for Filling Out:

Please rate the importance of the evaluation indicators according to the following scale:

| Very important | Important | Moderately important | Not important | Not at all important |
| --- | --- | --- | --- | --- |
| 5 | 4 | 3 | 2 | 1 |

2、Please rate the importance of the following indicators according to the above scale and fill in the "Importance Score" column. If you have any adjustment opinions on the following indicators, you may write them in the "Modification Suggestions" column. Thank you for your valuable opinions!

**First-Level Indicators**

| Indicator Content | Very important | Important | Moderately important | Not important | Not at all important | Modification Suggestions |
| --- | --- | --- | --- | --- | --- | --- |
| X1Policy Timeliness |  |  |  |  |  |  |
| X2Policy Evaluation |  |  |  |  |  |  |
| X3Incentives and Constraints |  |  |  |  |  |  |
| X4Policy Domains |  |  |  |  |  |  |
| X5 Policy Target Audiences |  |  |  |  |  |  |
| X6 Policy Perspectives |  |  |  |  |  |  |
| X7 Policy Priorities |  |  |  |  |  |  |
| Additional First-Level Indicators |  | | | | | |

**Second-Level Indicators**

| Indicator Content | Very important | Important | Moderately important | Not important | Not at all important | Modification Suggestions |
| --- | --- | --- | --- | --- | --- | --- |
| X1:1 long-term |  |  |  |  |  |  |
| X1:2 medium-term |  |  |  |  |  |  |
| X1:3 short-term |  |  |  |  |  |  |
| X2:1 clarity |  |  |  |  |  |  |
| X2:2compatibility |  |  |  |  |  |  |
| X2:3phased implementation |  |  |  |  |  |  |
| X2:4 quantifiable |  |  |  |  |  |  |
| X3:1financial guarantee |  |  |  |  |  |  |
| X3:2regulatory penalties |  |  |  |  |  |  |
| X3:3incentive system |  |  |  |  |  |  |
| X3:4oversight accountability |  |  |  |  |  |  |
| X3:5talent incentives |  |  |  |  |  |  |
| X4:1professional football |  |  |  |  |  |  |
| X4:2campus football |  |  |  |  |  |  |
| X4:3social football |  |  |  |  |  |  |
| X5:1football association |  |  |  |  |  |  |
| X5:2football club |  |  |  |  |  |  |
| X5:3youth |  |  |  |  |  |  |
| X5:4manager |  |  |  |  |  |  |
| X5:5training camp |  |  |  |  |  |  |
| X6:1macro |  |  |  |  |  |  |
| X6:2meso-level |  |  |  |  |  |  |
| X6:3micro |  |  |  |  |  |  |
| X7:1competition Results |  |  |  |  |  |  |
| X7:2cultivation of talent |  |  |  |  |  |  |
| X7:3mass participation |  |  |  |  |  |  |
| X7:4institutional reform |  |  |  |  |  |  |
| X7:5league adjustment |  |  |  |  |  |  |
| X7:6Construction of football fields |  |  |  |  |  |  |
| Additional Second-Level Indicators |  | | | | | |

*This questionnaire ends here. Please check if any items are missing.

*Thank you very much for your support and cooperation. Wish you a pleasant work and life!

Appendix 2

Research on the Evaluation of Chinese Football Reform Policies Based on the TA-PMC Model (First Round)

Dear Coaches/Experts:

Hello！

This study aims to screen and determine the core indicators for evaluating Chinese football reform policies through expert opinions, laying the foundation for subsequent analysis of policy evolution characteristics and efficacy. Your professional insights are crucial to this study, and we sincerely appreciate your support!

Now, please rate the importance of the indicators based on your actual situation and thoughts, and mark your scores in the corresponding options. If you have any new indicators or opinions, please also propose them; if there are no modifications, you may leave them blank.

**Part I: Basic Information of Experts**

1、Name：____

2、Gender：_____

3、Work Unit：_____

4、Years of Teaching or Training Experience：_____

5、Professional Title：_____

6、Research Direction：_____

**Part II: Screening of Evaluation Indicators for Football Reform Policies**

1、Instructions for Filling Out:

Please rate the importance of the evaluation indicators according to the following scale:

| Very important | Important | Moderately important | Not important | Not at all important |
| --- | --- | --- | --- | --- |
| 5 | 4 | 3 | 2 | 1 |

2、Please rate the importance of the following indicators according to the above scale and fill in the "Importance Score" column. If you have any adjustment opinions on the following indicators, you may write them in the "Modification Suggestions" column. Thank you for your valuable opinions!

**First-Level Indicators**

| Indicator Content | Very important | Important | Moderately important | Not important | Not at all important | Modification Suggestions |
| --- | --- | --- | --- | --- | --- | --- |
| X1Policy Timeliness |  |  |  |  |  |  |
| X2Policy Evaluation |  |  |  |  |  |  |
| X3Incentives and Constraints |  |  |  |  |  |  |
| X4Policy Domains |  |  |  |  |  |  |
| X5 Policy Target Audiences |  |  |  |  |  |  |
| X6 Policy Perspectives |  |  |  |  |  |  |
| X7 Policy Priorities |  |  |  |  |  |  |
| X8policy characteristics |  |  |  |  |  |  |
| X9 policy objectives |  |  |  |  |  |  |
| Additional First-Level Indicators |  | | | | | |

**Second-Level Indicators**

| Indicator Content | Very important | Important | Moderately important | Not important | Not at all important | Modification Suggestions |
| --- | --- | --- | --- | --- | --- | --- |
| X1:1 long-term |  |  |  |  |  |  |
| X1:2 medium-term |  |  |  |  |  |  |
| X1:3 short-term |  |  |  |  |  |  |
| X2:1 clarity |  |  |  |  |  |  |
| X2:2compatibility |  |  |  |  |  |  |
| X2:3phased implementation |  |  |  |  |  |  |
| X2:4 quantifiable |  |  |  |  |  |  |
| X3:1financial guarantee |  |  |  |  |  |  |
| X3:2regulatory penalties |  |  |  |  |  |  |
| X3:3incentive system |  |  |  |  |  |  |
| X3:4oversight accountability |  |  |  |  |  |  |
| X3:5talent incentives |  |  |  |  |  |  |
| X4:1professional football |  |  |  |  |  |  |
| X4:2campus football |  |  |  |  |  |  |
| X4:3social football |  |  |  |  |  |  |
| X5:1football association |  |  |  |  |  |  |
| X5:2football club |  |  |  |  |  |  |
| X5:3youth |  |  |  |  |  |  |
| X5:4manager |  |  |  |  |  |  |
| X5:5training camp |  |  |  |  |  |  |
| X6:1macro |  |  |  |  |  |  |
| X6:2meso-level |  |  |  |  |  |  |
| X6:3micro |  |  |  |  |  |  |
| X7:1competition Results |  |  |  |  |  |  |
| X7:2cultivation of talent |  |  |  |  |  |  |
| X7:3mass participation |  |  |  |  |  |  |
| X7:4institutional reform |  |  |  |  |  |  |
| X7:5league adjustment |  |  |  |  |  |  |
| X7:6Construction of football fields |  |  |  |  |  |  |
| X8:1announcement-oriented |  |  |  |  |  |  |
| X8:2predictive modeling |  |  |  |  |  |  |
| X8:3supportive |  |  |  |  |  |  |
| X8:4planning-oriented |  |  |  |  |  |  |
| X8:5regulatory |  |  |  |  |  |  |
| X9:1competitive performance |  |  |  |  |  |  |
| X9:2cultural atmosphere |  |  |  |  |  |  |
| X9:3Number of football Fields |  |  |  |  |  |  |
| Additional Second-Level Indicators |  | | | | | |

*This questionnaire ends here. Please check if any items are missing.

*Thank you very much for your support and cooperation. Wish you a pleasant work and life!

Appendix 3

Research on the Evaluation of Chinese Football Reform Policies Based on the TA-PMC Model (Third Round)

Dear Coaches/Experts:

Hello！

This study aims to screen and determine the core indicators for evaluating Chinese football reform policies through expert opinions, laying the foundation for subsequent analysis of policy evolution characteristics and efficacy. Your professional insights are crucial to this study, and we sincerely appreciate your support!

Now, please rate the importance of the indicators based on your actual situation and thoughts, and mark your scores in the corresponding options. If you have any new indicators or opinions, please also propose them; if there are no modifications, you may leave them blank.

**Part I: Basic Information of Experts**

1、Name：____

2、Gender：_____

3、Work Unit：_____

4、Years of Teaching or Training Experience：_____

5、Professional Title：_____

6、Research Direction：_____

**Part II: Screening of Evaluation Indicators for Football Reform Policies**

1、Instructions for Filling Out:

Please rate the importance of the evaluation indicators according to the following scale:

| Very important | Important | Moderately important | Not important | Not at all important |
| --- | --- | --- | --- | --- |
| 5 | 4 | 3 | 2 | 1 |

2、Please rate the importance of the following indicators according to the above scale and fill in the "Importance Score" column. If you have any adjustment opinions on the following indicators, you may write them in the "Modification Suggestions" column. Thank you for your valuable opinions!

**First-Level Indicators**

| Indicator Content | Very important | Important | Moderately important | Not important | Not at all important | Modification Suggestions |
| --- | --- | --- | --- | --- | --- | --- |
| X1Policy Timeliness |  |  |  |  |  |  |
| X2Policy Evaluation |  |  |  |  |  |  |
| X3Incentives and Constraints |  |  |  |  |  |  |
| X4Policy Domains |  |  |  |  |  |  |
| X5 Policy Target Audiences |  |  |  |  |  |  |
| X6 Policy Perspectives |  |  |  |  |  |  |
| X7 Policy Priorities |  |  |  |  |  |  |
| X8policy characteristics |  |  |  |  |  |  |
| X9 policy objectives |  |  |  |  |  |  |
| Additional First-Level Indicators |  | | | | | |

**Second-Level Indicators**

| Indicator Content | Very important | Important | Moderately important | Not important | Not at all important | Modification Suggestions |
| --- | --- | --- | --- | --- | --- | --- |
| X1:1 long-term |  |  |  |  |  |  |
| X1:2 medium-term |  |  |  |  |  |  |
| X1:3 short-term |  |  |  |  |  |  |
| X2:1 clarity |  |  |  |  |  |  |
| X2:2compatibility |  |  |  |  |  |  |
| X2:3phased implementation |  |  |  |  |  |  |
| X2:4 quantifiable |  |  |  |  |  |  |
| X3:1financial guarantee |  |  |  |  |  |  |
| X3:2regulatory penalties |  |  |  |  |  |  |
| X3:3incentive system |  |  |  |  |  |  |
| X3:4oversight accountability |  |  |  |  |  |  |
| X3:5talent incentives |  |  |  |  |  |  |
| X4:1professional football |  |  |  |  |  |  |
| X4:2campus football |  |  |  |  |  |  |
| X4:3social football |  |  |  |  |  |  |
| X5:1football association |  |  |  |  |  |  |
| X5:2football club |  |  |  |  |  |  |
| X5:3youth |  |  |  |  |  |  |
| X5:4manager |  |  |  |  |  |  |
| X5:5training camp |  |  |  |  |  |  |
| X6:1macro |  |  |  |  |  |  |
| X6:2meso-level |  |  |  |  |  |  |
| X6:3micro |  |  |  |  |  |  |
| X7:1competition Results |  |  |  |  |  |  |
| X7:2cultivation of talent |  |  |  |  |  |  |
| X7:3mass participation |  |  |  |  |  |  |
| X7:4institutional reform |  |  |  |  |  |  |
| X7:5league adjustment |  |  |  |  |  |  |
| X7:6Construction of football fields |  |  |  |  |  |  |
| X8:1announcement-oriented |  |  |  |  |  |  |
| X8:2predictive modeling |  |  |  |  |  |  |
| X8:3supportive |  |  |  |  |  |  |
| X8:4planning-oriented |  |  |  |  |  |  |
| X8:5regulatory |  |  |  |  |  |  |
| X9:1competitive performance |  |  |  |  |  |  |
| X9:2cultural atmosphere |  |  |  |  |  |  |
| X9:3Robust mechanisms |  |  |  |  |  |  |
| X9:4Upgrading the population |  |  |  |  |  |  |
| X9:5Number of football Fields |  |  |  |  |  |  |
| Number of football Fields |  |  |  |  |  |  |
| Additional Secondary Indicators |  | | | | | |

*This questionnaire ends here. Please check if any items are missing.

*Thank you very much for your support and cooperation. Wish you a pleasant work and life!
