# Supplementary figures and images for: The decade of China’s football reform: Evolutionary characteristics, performance evaluation, and reflections and insights
Source: PLoS One. 2025 Dec 30;20(12):e0339264. doi: 10.1371/journal.pone.0339264 (PMC12753081; doi:10.1371/journal.pone.0339264)

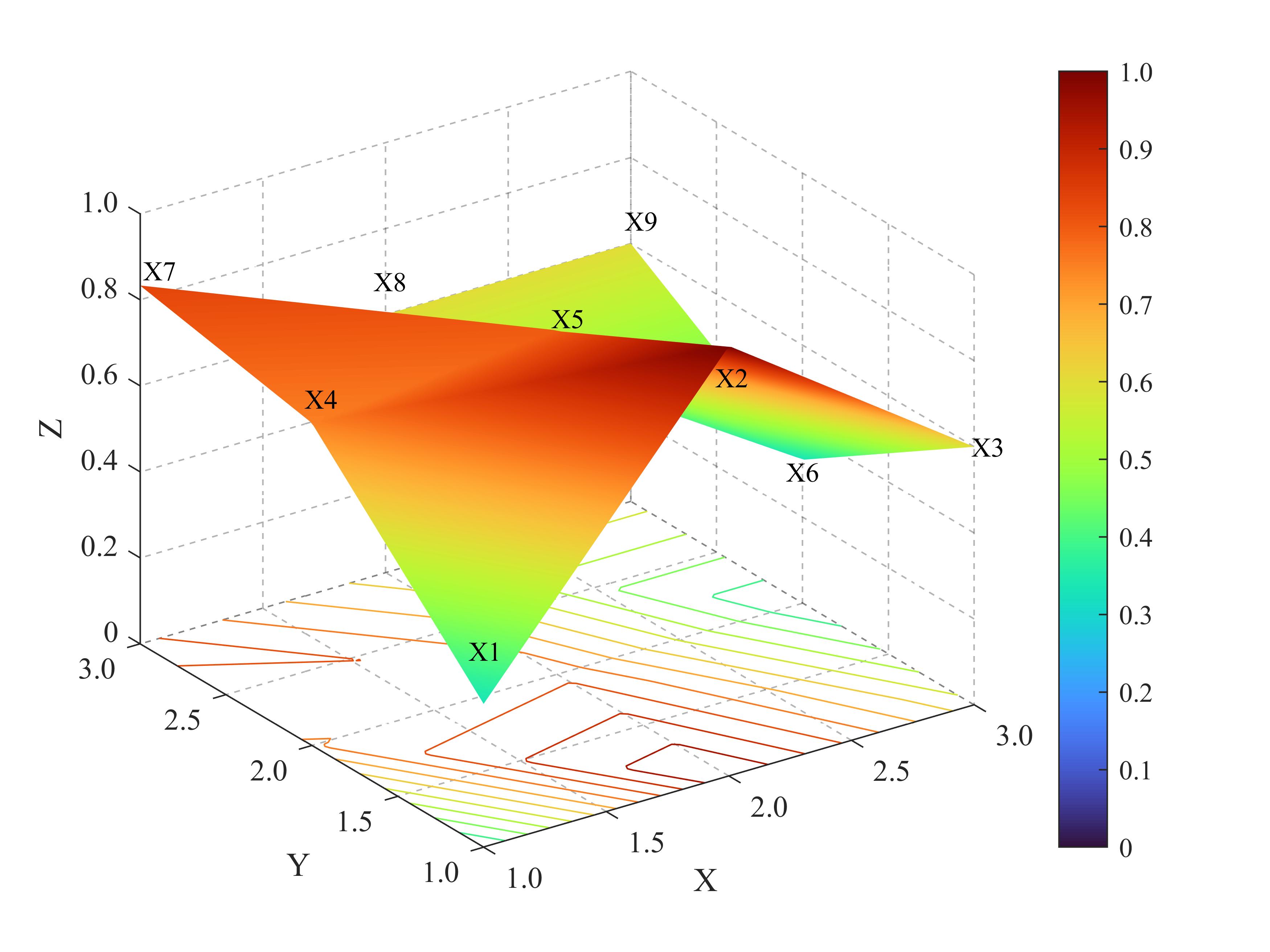

Supplement: S2 Source Data — (TAR) [file pone.0339264.s002.tar › Source Data/PMC surface chart/figures/P-8-1.jpg]

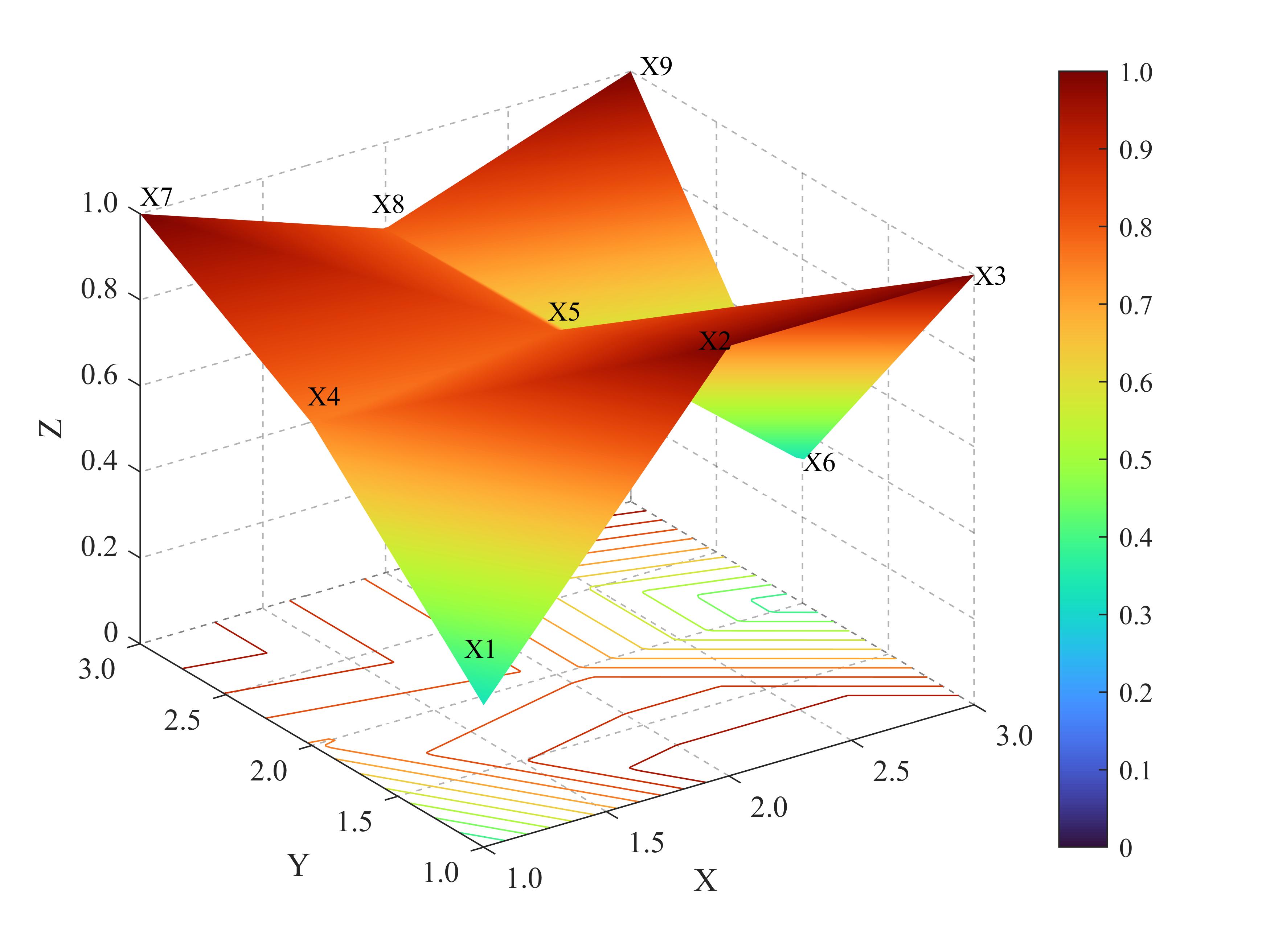

Supplement: S2 Source Data — (TAR) [file pone.0339264.s002.tar › Source Data/PMC surface chart/figures/P1-1.jpg]

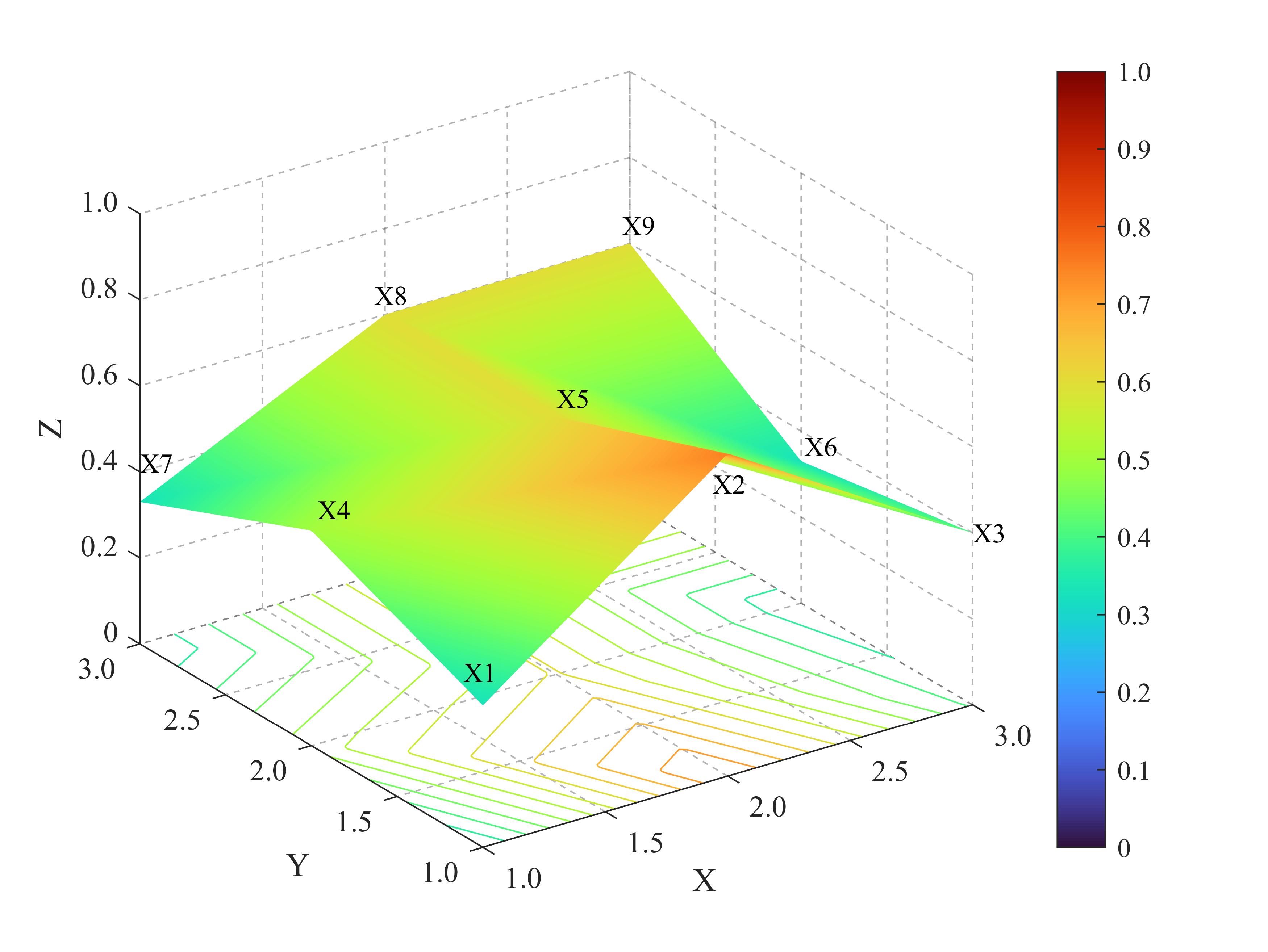

Supplement: S2 Source Data — (TAR) [file pone.0339264.s002.tar › Source Data/PMC surface chart/figures/P10-1.jpg]

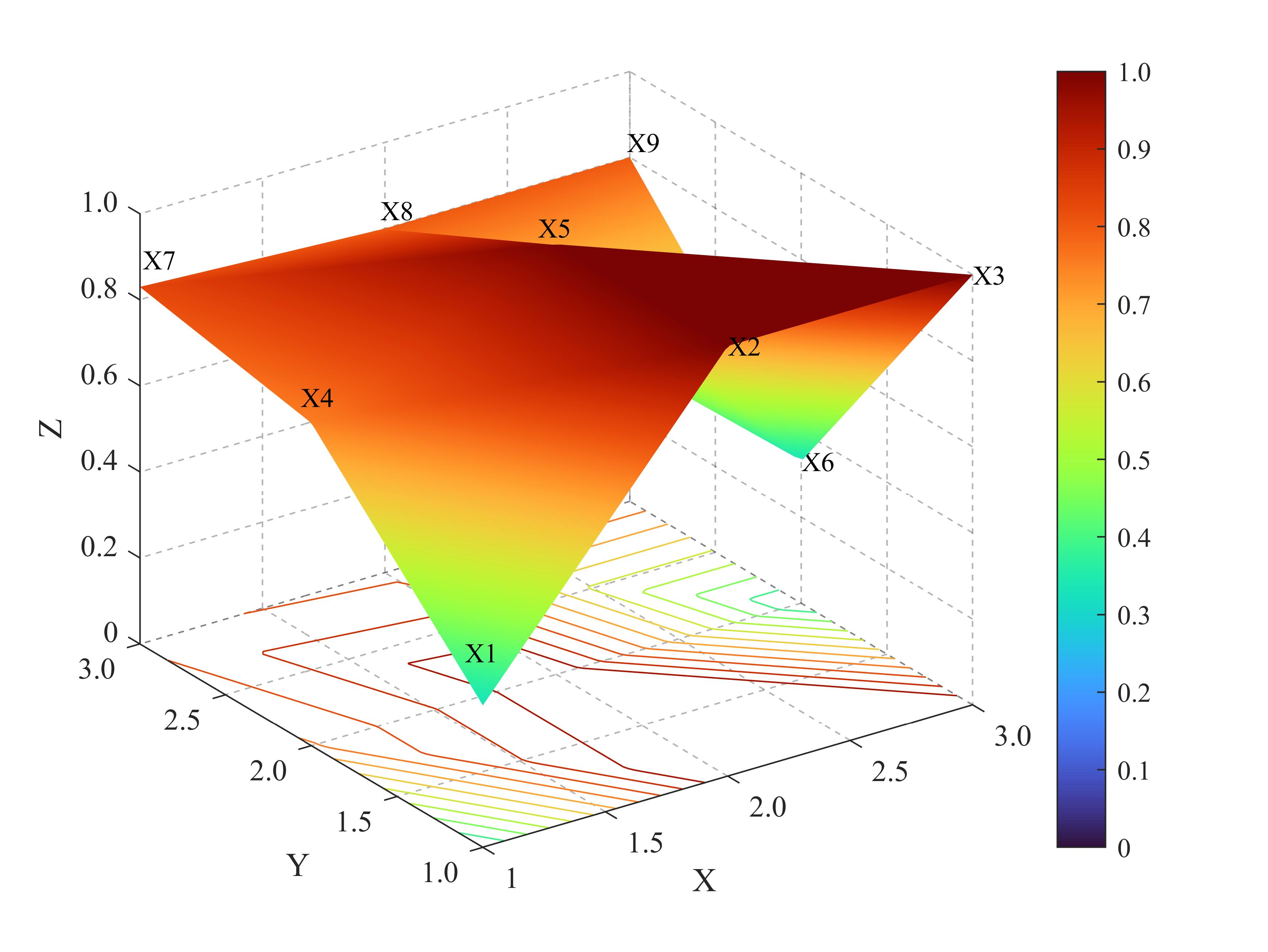

Supplement: S2 Source Data — (TAR) [file pone.0339264.s002.tar › Source Data/PMC surface chart/figures/P3-1.jpg]

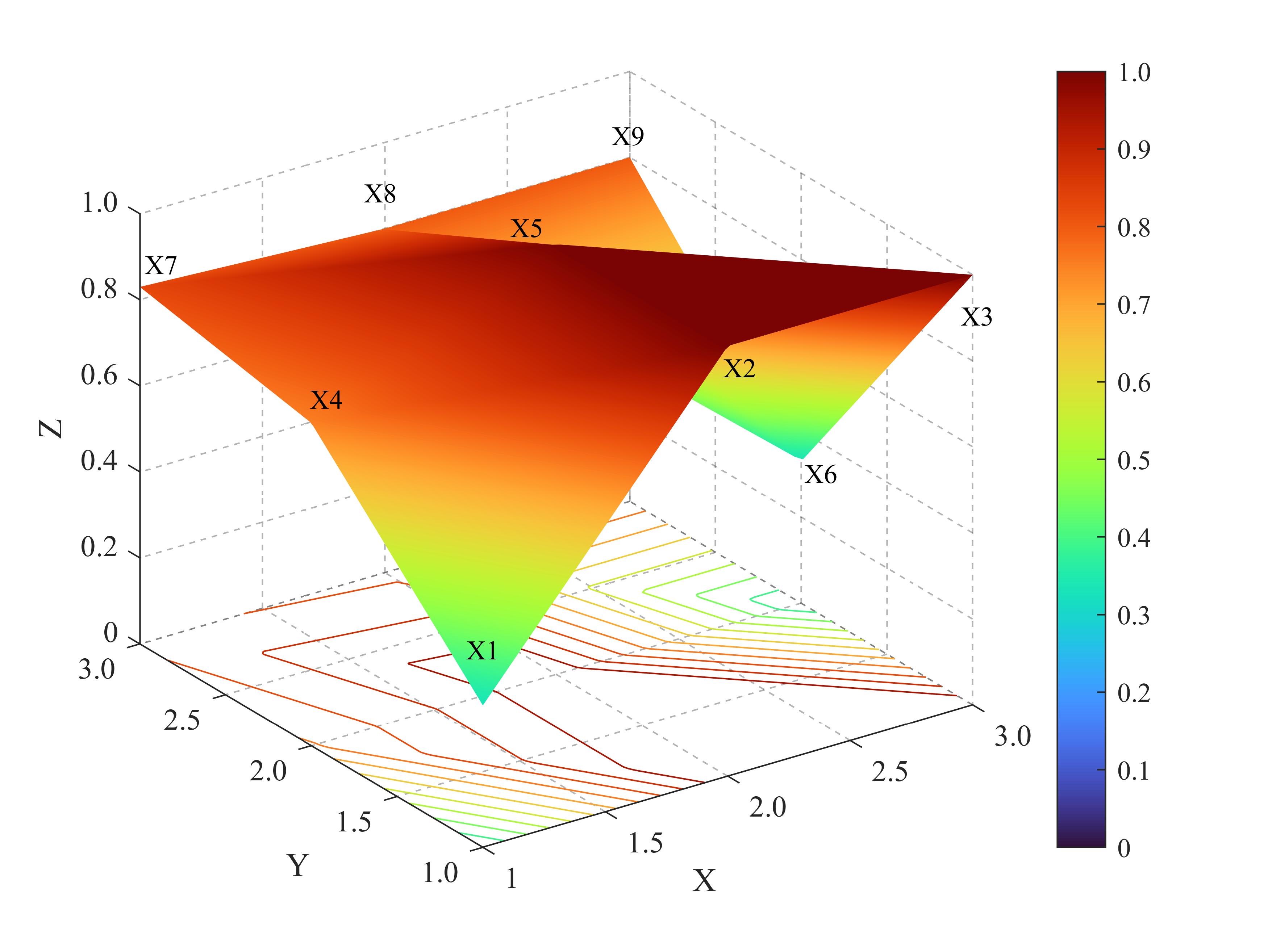

Supplement: S2 Source Data — (TAR) [file pone.0339264.s002.tar › Source Data/PMC surface chart/figures/P6-1.jpg]

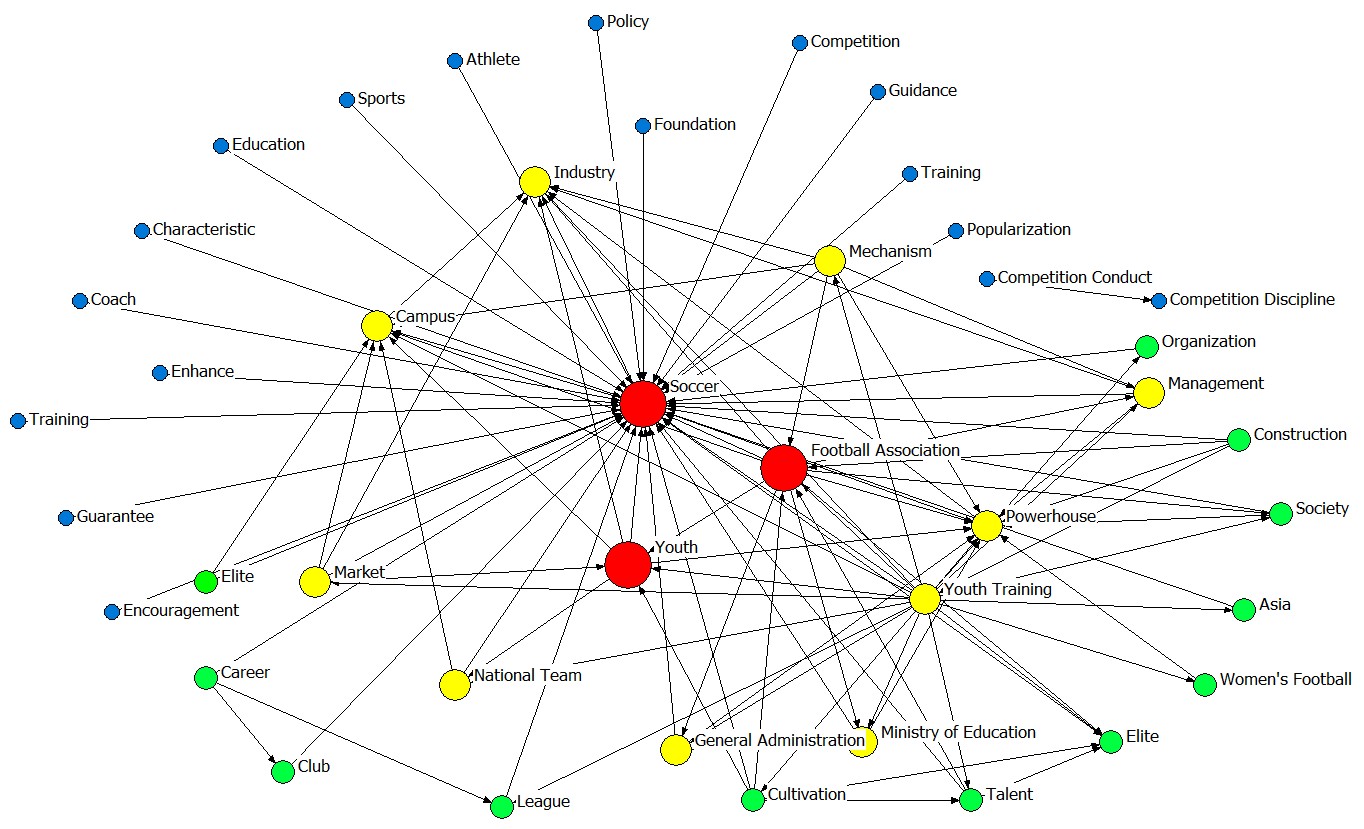

Supplement: S2 Source Data — (TAR) [file pone.0339264.s002.tar › Source Data/PMC surface chart/figures/Semantic Network figure.jpg]

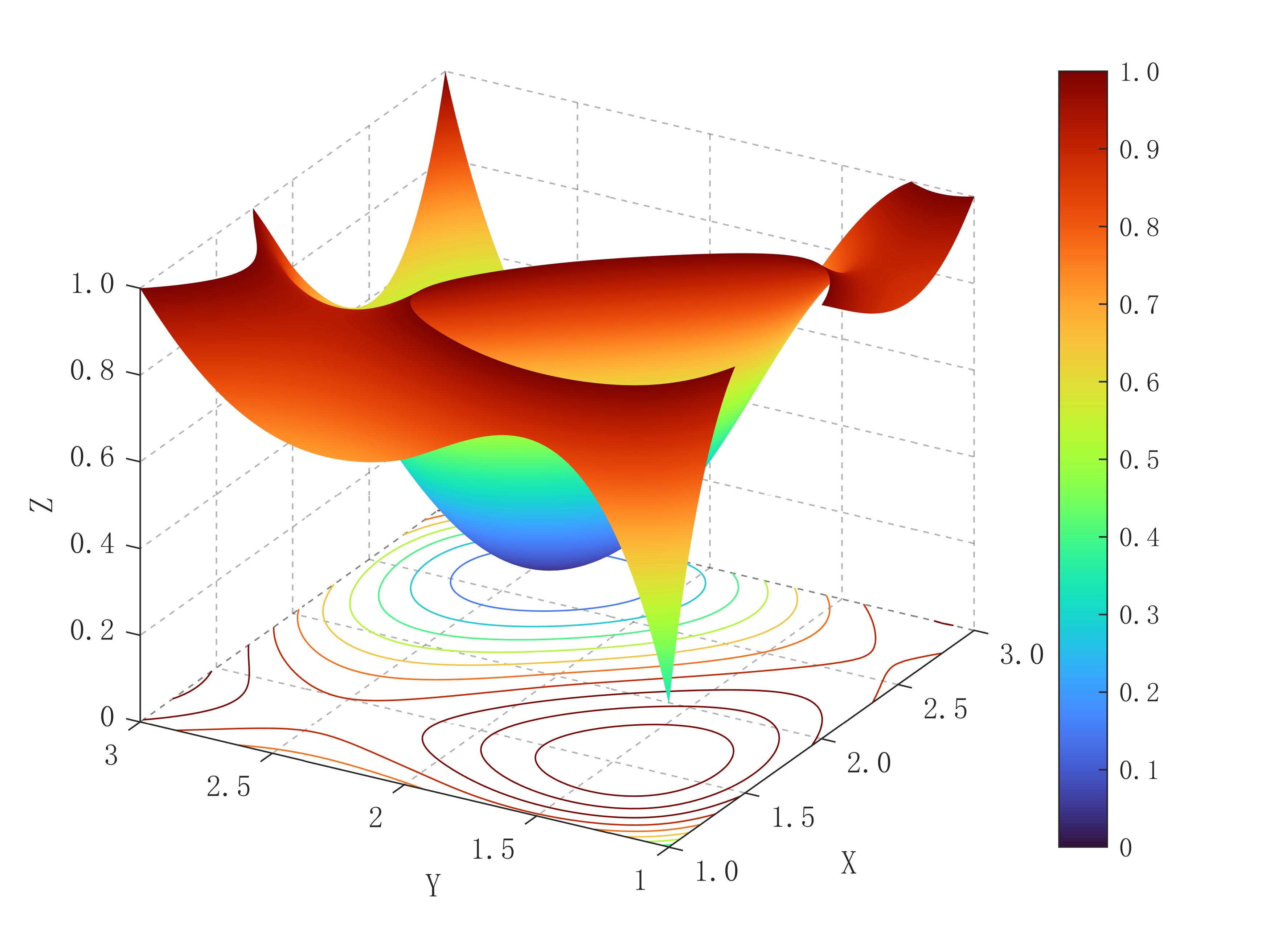

Supplement: S2 Source Data — (TAR) [file pone.0339264.s002.tar › Source Data/PMC surface chart/P1.jpg]

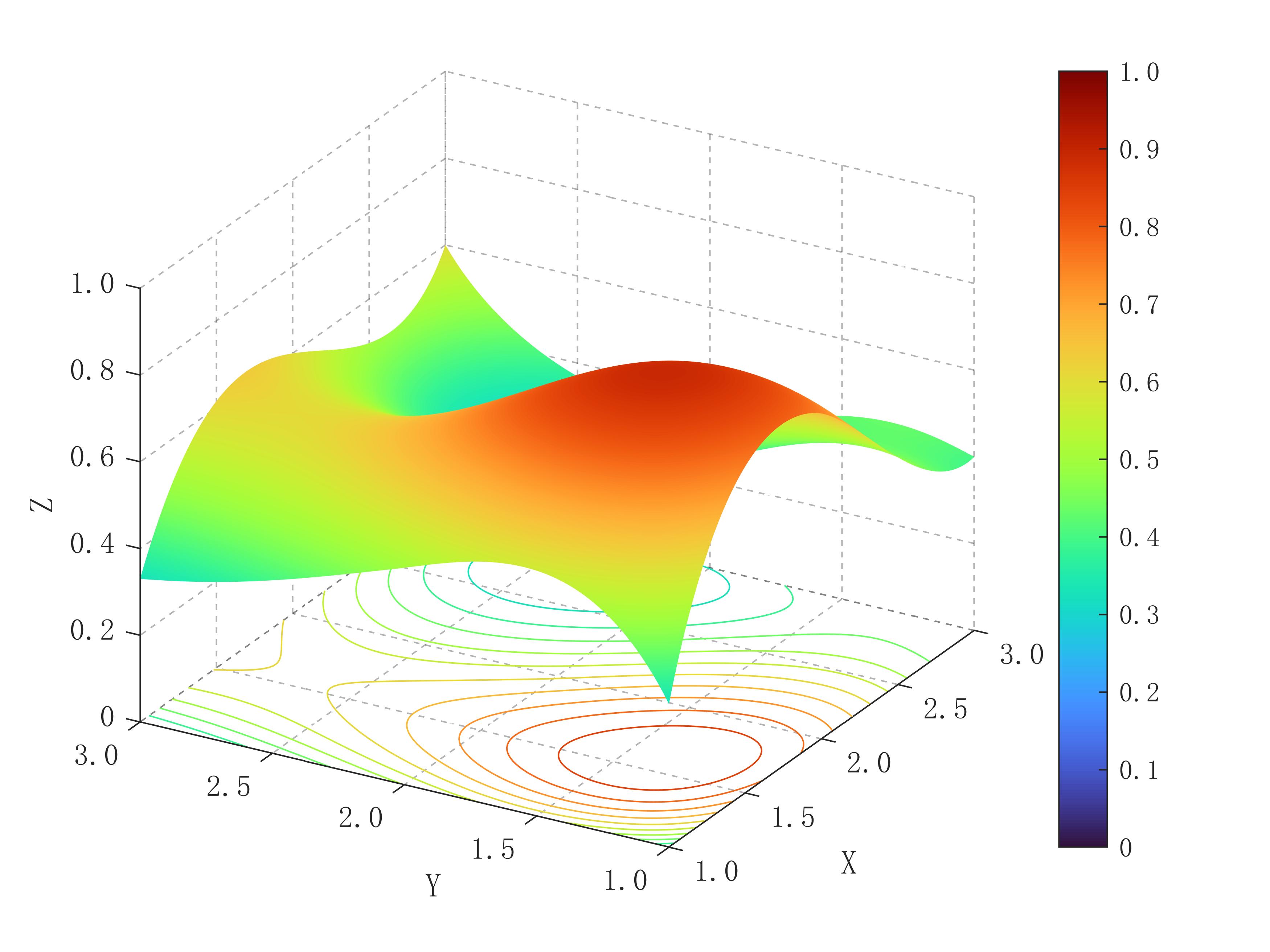

Supplement: S2 Source Data — (TAR) [file pone.0339264.s002.tar › Source Data/PMC surface chart/P10.jpg]

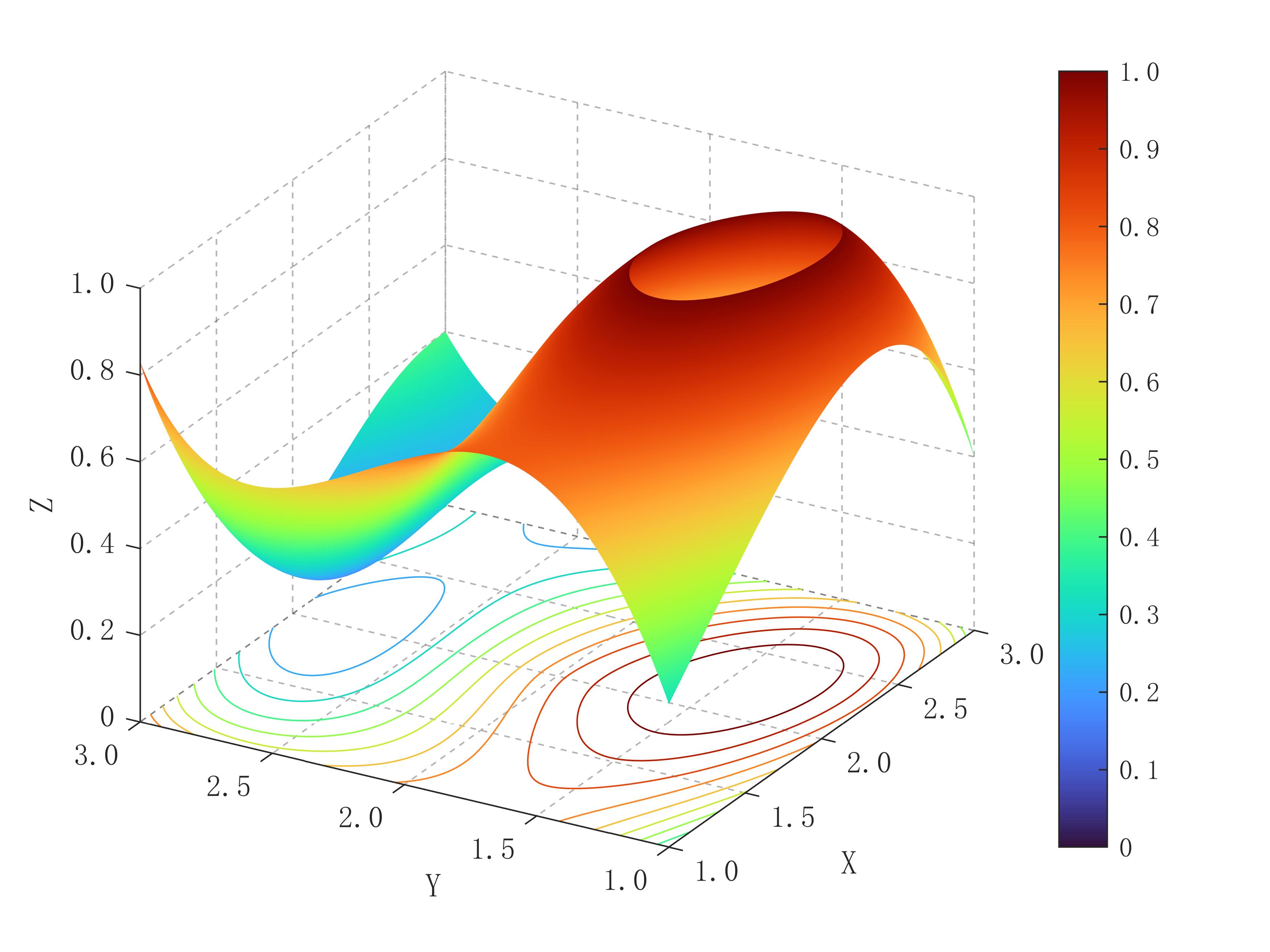

Supplement: S2 Source Data — (TAR) [file pone.0339264.s002.tar › Source Data/PMC surface chart/P6.jpg]
